# Tissue Extract from Brittle Star Undergoing Arm Regeneration Promotes Wound Healing in Rat

**DOI:** 10.3390/md21070381

**Published:** 2023-06-28

**Authors:** Alireza Afshar, Arezoo Khoradmehr, Fariborz Nowzari, Neda Baghban, Masoud Zare, Maryam Najafi, Seyedeh Zahra Keshavarzi, Fatemeh Zendehboudi, Gholamhossein Mohebbi, Alireza Barmak, Fatemeh Mohajer, Nahid Basouli, Mohammadreza Keshtkar, Aida Iraji, Fatemeh Sari Aslani, Cambyz Irajie, Iraj Nabipour, Mehdi Mahmudpour, Nader Tanideh, Amin Tamadon

**Affiliations:** 1 PerciaVista R&D Co., Shiraz 73, Iran; 2 Student Research Committee, Bushehr University of Medical Sciences, Bushehr 75, Iran; 3 The Persian Gulf Marine Biotechnology Research Center, The Persian Gulf Biomedical Sciences Research Institute, Bushehr University of Medical Sciences, Bushehr 75, Iran; 4 Stem Cells Technology Research Center, Shiraz University of Medical Sciences, Shiraz 73, Iran; 5 Food Lab, Bushehr University of Medical Sciences, Bushehr 75, Iran; 6 Medicinal and Natural Products Chemistry Research Center, Shiraz University of Medical Sciences, Shiraz 73, Iran; 7 Central Research Laboratory, Shiraz University of Medical Sciences, Shiraz 73, Iran; 8 Molecular Dermatology Research Center, School of Medicine, Shiraz University of Medical Sciences, Shiraz 73, Iran; 9 Department of Medical Biotechnology, School of Advanced Medical Sciences and Technologies, Shiraz University of Medical Sciences, Shiraz 73, Iran; 10 The Persian Gulf Tropical Medicine Research Center, The Persian Gulf Biomedical Sciences Research Institute, Bushehr University of Medical Sciences, Bushehr 75, Iran; 11 Department of Pharmacology, Medical School, Shiraz University of Medical Sciences, Shiraz 73, Iran; 12 Department for Scientific Work, West Kazakhstan Marat Ospanov Medical University, Aktobe 030012, Kazakhstan

**Keywords:** wound healing, regeneration, proliferation, brittle star, rat

## Abstract

This study set out to evaluate the wound healing properties of brittle star extracts in vitro and in vivo. Due to the great arm regeneration potential of the brittle star, *Ophiocoma cynthiae*, the present study aimed to evaluate the wound healing effect of hydroalcoholic extracts of brittle star undergoing arm regeneration in wound healing models. The brittle star samples were collected from Nayband Bay, Bushehr, Iran. After wound induction in the arm of brittle stars, hydroalcoholic extracts relating to different times of arm regeneration were prepared. The GC-MS analysis, in vitro MTT cell viability and cell migration, Western blot, and computational analysis tests were performed. Based on the in vitro findings, two BSEs were chosen for in vivo testing. Macroscopic, histopathological and biochemical evaluations were performed after treatments. The results showed positive proliferative effects of BSEs. Specifically, forty-two compounds were detected in all groups of BSEs using GC-MS analysis, and their biological activities were assessed. The MTT assay showed that the 14 d BSE had a higher proliferative effect on HFF cells than 7 d BSE. The cell migration assay showed that the wound area in 7 d and 14 d BSEs was significantly lower than in the control group. Western blot analysis demonstrated an increase in the expression of proliferation-related proteins. Upon the computational analysis, a strong affinity of some compounds with proteins was observed. The in vivo analysis showed that the evaluation of wound changes and the percentage of wound healing in cell migration assay in the 7 d BSE group was better than in the other groups. Histopathological scores of the 7 d BSE and 14 d BSE groups were significantly higher than in the other groups. In conclusion, the hydroalcoholic extract of *O. cynthiae* undergoing arm regeneration after 7 and 14 days promoted the wound healing process in the cell and rat skin wound healing model due to their proliferative and migratory biological activity.

## 1. Introduction

The skin is the largest organ in the mammalian body [1]. An important task of the skin is to create a defense barrier against external and infectious agents [2]. Loss of skin surface integrity allows microbial invasion of the body as well as the development of various diseases [3]. In the case of loss of integrity due to wounds to the skin, the skin must undergo a regeneration and healing process. The wound healing process has been divided into four stages, including the hemostasis, inflammation, proliferation, and remodeling stages, which overlap with each other [4]. This process of healing in mammals takes time and is imperfect [5]. In some cases, wounds can become chronic, and if the damage is severe, this can lead to death [6]. Some injuries, such as some types of burn wounds, take a long time to heal [7], and thus need to be cured more quickly. Several factors such as insufficient blood supply, external pressure on the wound, and diabetes can cause the wound to become chronic or even not healable [8]. Furthermore, wounds that have not responded to treatment after more than a year are more likely to be incurable [9]. In addition, mammalian wound healing is usually accompanied by scar tissue formation, which is an imperfection in mammalian tissue regeneration [10]. Previous studies have classified wound healing methods into two classes: conventional processes and regenerative processes. The difference between these methods is scar formation. In the conventional method, scar tissue will eventually form; a fibrotic tissue that causes part of the skin to be aesthetically and functionally different [11,12]. However, regenerative treatment results in perfect regeneration of damaged skin without scar formation [12]. The current study focuses on regenerative treatments for skin tissue with an emphasis on the absence of scar formation. Some materials that are used in wound healing and other wound dressing methods can be extracted from plants, animals, and other living organisms or minerals [13].

Most marine invertebrates have enormous regenerative capabilities. The use of marine invertebrates in the treatment of wounds has attracted the attention of researchers [14]. These capabilities can be seen in the Echinodermata phylum [15]. In this phylum, there are many species, such as brittle stars, that they can undergo regeneration in almost any part of their body [16]. A specific type of brittle star called *Ophiocoma cynthiae*, which can regenerate the entirety of its arm, can be found widely in the Persian Gulf [16]. 

The stages of arm regeneration in different brittle star species are almost the same, but the time of regeneration can be different [16,17]. In the brittle star species *Ophioderma longicaudum*, the process of arm regeneration starts with the protection of the area with external skeletal shields, which close the wound area within 1 to 3 days [17]. Subsequently, a regenerative bud forms, and at this stage, cell migration and epithelial cell hypertrophy occur in order to close the wounded area [17]. Furthermore, a large number of phagocyte and small cells take part in the regeneration process [17]. One week after arm amputation, the wound area appears to be healing and is covered by a thick cicatricial layer including a large number of small cells and phagocytes [17,18]. During the second week of arm regeneration, blastema formation occurs [17]. At this stage, some canals start regrowing in association with the numbers of mitotic cells distributed in the mesenchymal layer [17]. Blastema regeneration increases during week 3, and coelomic components can be clearly identified [17]. The full process of arm regeneration in *O. longicaudum* reaches completion in week 12. Almost the same arm regeneration process, with some variations, has been reported for *Amphiura filiformis* [16,17]. The time of arm regeneration in this species is 24 days, with blastema formation occurring 3 days after arm amputation [16,17]. The authors showed that blastema develops between 4 and 6 days after arm amputation, and the regeneration process completes its final stages and reconstruction between 16 and 24 days post amputation [16,17].

Several studies have shown that extracts from the Echinodermata phylum include diverse bioactive compounds. Brittle star extracts (BSEs) contain various compounds that exert different biological effects in vitro and in vivo. Extracts from starfish, another species from the Echinodermata phylum, has many bioactive substances and has been shown to be effective at all stages of wound healing in a zebrafish wound model [19]. Additionally, it has been reported that the starfish extract mediates both the growth process of tissue and the overgrowth of tissue, thus preventing scar tissue formation [19]. Furthermore, the various bioactive compounds extracted from brittle stars exerted anti-cancer, antioxidant, anti-inflammatory, and proliferative effects [20,21,22,23,24,25,26]. Methanolic extract of *O. erinaceus* exerts antioxidant effects on A2780cp ovarian cancer cells [24]. Another study demonstrated that the saponin fraction of *O. erinaceus* exerts anti-inflammatory effects on human THP-1 monocytes cells [26]. Additionally, the saponin fraction of *O. erinaceus* has been shown to exert a metastatic inhibitory effect [22]. Moreover, several studies have shown that the methanol extract of *O. erinaceus* has antiproliferative and pro-apoptotic effects on human cancer cells in vitro [21,23,25]. In contrast, polysaccharides extracted from *O. erinaceus* were shown to have a positive effect on a rat wound healing model [20]. Additionally, another study on *O. erinaceus* whole-body alcoholic extract showed that it was able to increase wound healing process in a mouse wound healing model [27]. However, to the best of our knowledge, the effects of bioactive substances produced during different stages of arm regeneration in brittle stars, including *O. cynthiae*, have not been evaluated with respect to wound healing on mammalian skin, either in vitro or in vivo. Considering the anti-inflammatory, antioxidant and proliferative effects of Echinodermata phylum extracts on wound healing [19,24,26,27], the purposes of the present study was to evaluate the effects of a crude extract of *O. cynthiae* undergoing arm regeneration on an in vitro, a computational model, and an in vivo study (a wide wound excision in male rats).

## 2. Results

### 2.1. Species Identification Demonstrated That the Persian Gulf Brittle Star Was Ophiocoma Cynthiae

This examination led to the identification of *Ophiocoma cynthiae* (Figure S1) from the Family Ophiocomidae, order Ophiurida and Genus Ophiocoma. In terms of the features of the brittle star, the disc diameter was 17 mm (Figure S1). The form of the disc was pentagonal, with slightly incised interradial margins [28]. The aboral side of the disc and arms were uniformly black. The dorsal disc was covered densely with spherical granules [28] (Figure S2A), covering the whole surface, including the radial shields, which could not be distinguished. A few granules were sparsely distributed along the genital slit on the oral surface [28] (Figure S2B). There were five simple arms. The spines were cylindrical in shape [29] (Figure S2C). Two scales of tentacle existed per podial pore, which were oval in shape and equivalent in size [28,30] (Figure S2E). Three oral papillae were observed on each side of the jaw angle, with the distal-most oral papillae being rectangular in shape [30]. Additionally, there were three clusters of small tooth papillae on the top of each jaw [28]. The edges of oral and tooth papillae were smooth (7). The oral shields were oval-shaped, longer than they were broad, and the widest point a little toward the distal direction [28] (Figure S2D). The color of the feet tube was grey. The triangular aboral shields were much smaller than the oral shield [28]. The aboral arm plates were oval to trapezoidal in shape, and were broadly in contact with one another [30], with a proximal concave margin and a distal convex margin (Figure S2F).

### 2.2. Amputated Arm of O. cynthiae Regenerated in 14 d

**Figure 1:**
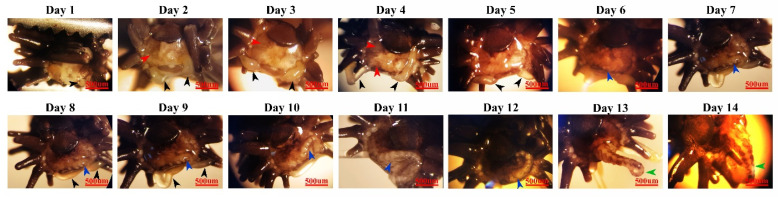
Macroscopic evaluation of brittle star arm regeneration over 14 days. The black arrow indicates spine bud formation and growth. The red arrow indicates re-epithelialization. The blue arrow indicates arm bud formation and regeneration. The green arrow indicates the complete small-scale formation of a new brittle star arm. Scale bars are 500 µm.

The arm regeneration process was evaluated macroscopically (Figure 1). The wound area and the exposed internal coelomic canal of the amputated arm of brittle stars were completely closed at the end of day 1 (Figure 1). It seems that at this stage, by means of muscle contraction and arm shields, the wound area was mostly closed in order to protect the arm. Early on day 1, two regenerating buds started to form symmetrically on the ventral side of the wound area. These two buds formed the new spines of the regenerating arm. Subsequently, in order to completely close the wound area, re-epithelialization occurred early on day 2. Additionally, during the early stages of days 6 and 7, another regenerative bud formed at the central part of the wound area. This bud grew longitudinally grown, and was ultimately refashioned into a new brittle star arm on day 14. During this regeneration, no scar tissue formation took place on the brittle stars’ arm.

**Figure 2:**
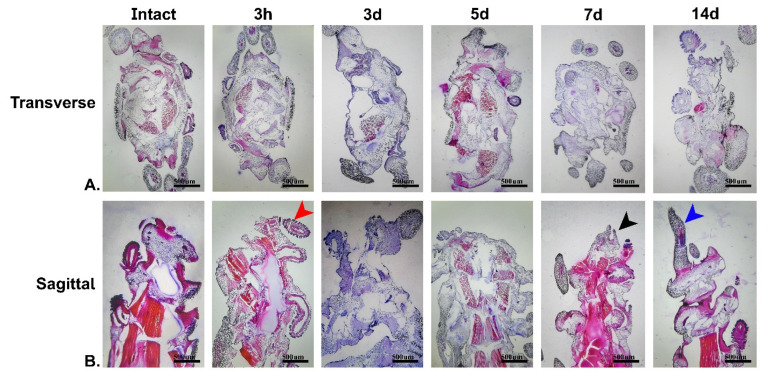
Microscopic evaluation of brittle star arm regeneration over 14 days (**A**) Transverse; (**B**) Sagittal. The red arrow indicates spine bud formation. The black arrow indicates arm bud for-mation and regeneration. The blue arrow indicates the complete formation of a new brittle star arm. Scale bars are 500 µm.

In addition, the arm regeneration process of the *O. cynthiae* was tracked histologically (Figure 2). The histological images proved the occurrence of some changes described previously on the basis of macroscopic images. Specifically, the formation of the regenerating bud at hour three is shown. In addition, the formation of the regenerative bud on day 7 and the formation of the new arm on day 14 are shown (Figure 2).

### 2.3. GC-MS Profile of O. cynthiae Extract Showed Proliferative and Migrative Compounds in 7d and 14d BSE Extracts

The GC-MS profile showed different compounds with various biological activities (Figures S3–S7). In total, there were 42 compounds in the GC-MS profile of the hydroalcoholic extract of *O. cynthiae* taken at different times. The 0 h, 3 h, 3 d, 5 d, 7 d, and 14 d extracts of the brittle star contained 22, 3, 10, 8, 9, and 9 compounds, respectively (Table 1). Specifically, there were three potent compounds with proliferative and regenerative effects: cholesterol, pseduosarsasapogenin-5,20-dien methyl ether, and ethyl.alpha-d-glucopyranoside, which were detected in the 0 h and 7 d BSE, the 0 h and 3 d BSE, and the 7 d BSE, respectively. Moreover, octadecanoic acid, methyl ester (methyl stearate), the cell migratory effect of which was described in a previous study (Table 1), was observed in the 14 d extract. In addition, other compounds with different biological activities, including anti-inflammatory, antimicrobial, anti-proliferation, antioxidant, and antifibrotic activities, were observed in the extracts (Table 1). The GC-MS analysis showed that the 7 d and 14 d BSEs had cell proliferation- and migration-induced compounds, respectively; in vitro analyses were performed to evaluate the biological properties of the 7 d and 14 d BSEs and to compare them with the other BSEs.

### 2.4. The 7 d and 14 d BSEs Did Not Have Negative Effects on Cell Viability

**Figure 3:**
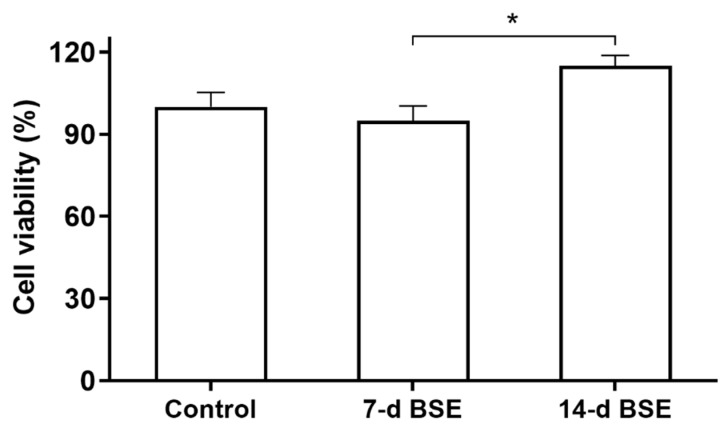
Human foreskin fibroblast (HFF) viability ratio after 72 h exposure to 7 d and 14 d brittle star extracts (BSEs) undergoing arm regeneration, using MTT assay. * *p* < 0.05.

The MTT assay showed that the 14 d BSE had a higher viability ratio on HFFs than 7 d BSE (*p* = 0.03, Figure 3). There were no statistically significant differences in cell viability among HFFs receiving the 7 d and 14 d BSE treatments and the control treatment.

### 2.5. In Vitro Cell Migration Assay of Brittle Star Extract Showed the Migratory Effects of the 7 d and 14 d Extracts

**Figure 4:**
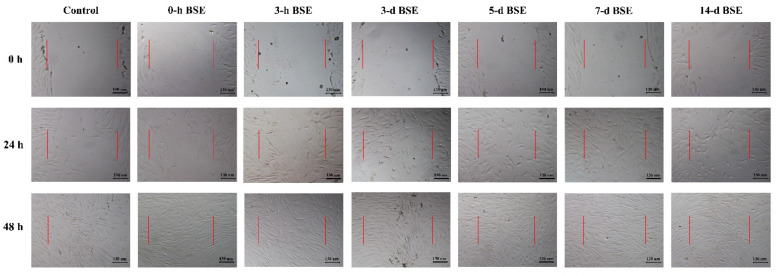
In vitro analysis of brittle star extracts (BSEs). Wound area in cell culture wound healing model treated with different BSEs for a total of 48 h. Scale bars are 130 µm. Red lines show primary edges of induced wounds in cell culture model.

Based on the MTT assay results, the 7 d and 14 d BSEs were chosen for further analysis. The HFFs wound area was closed completely by 48 h in all groups (Figure 4). Visually, the wound areas of the cells receiving treatments with different extracts were visually distinguishable at 24 h (Figure 4). The wound area in the cell culture model was calculated using ImageJ software 0, 24 and 48 h after wound induction.

**Figure 5:**
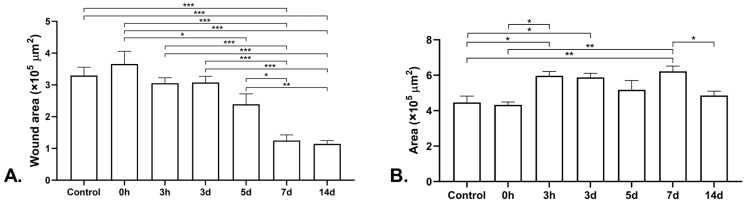
In vitro cell migration assay of brittle star extracts. (**A**) Wound area in cell culture wound healing model after 24 h. (**B**) Subtraction of wound area at 0 h from that at 24 h in cell culture wound healing model. * *p* < 0.05, ** *p* < 0.01 and *** *p* < 0.001.

An analysis of the image displaying the results of the 24 h cell migration assay showed that the wound area in the control group was higher than that in the groups receiving the 7 d and 14 d BSE treatment at 24 h (*p* < 0.001, Figure 5A). There were no significant differences between the control group and the other groups. Additionally, the wound area in the group receiving the 0 h BSE was higher than those in the groups receiving the 5, 7 and 14 d BSEs (*p* = 0.019, *p* < 0.001 and *p* < 0.001, respectively, Figure 5A). Moreover, treatment with the 3 h and 3 d BSEs resulted in a higher wound area than treatment with the 7 and 14 d extracts (*p* < 0.001, Figure 5A). Furthermore, treatment with the 5 d BSE resulted in a wound area larger than that obtained when treated with the 7 and 14 d extracts (*p* = 0.028 and *p* = 0.006, respectively, Figure 5A). There was no difference between the 7 d and 14 d BSEs with respect to the wound area. The wound area at 0 h was subtracted from that at 24 h for all extract groups (Figure 5B). The subtraction area when treated with the 3 h extract was higher than that of the control group and 0 h BSE group (*p* = 0.019 and *p* = 0.025, respectively, Figure 5B). Moreover, this subtraction area in the 3 d BSE group was larger than that of the control group (*p* = 0.046, Figure 5B). Additionally, the subtraction area was higher for the 7 d BSE group than for the control group and the 0 h and 14 d BSE groups (*p* = 0.007, *p* = 0.009 and *p* = 0.046, respectively, Figure 5B).

### 2.6. Western Blot Profile of O. cynthiae Extracts Showed Different Expression Proteins

**Figure 6:**
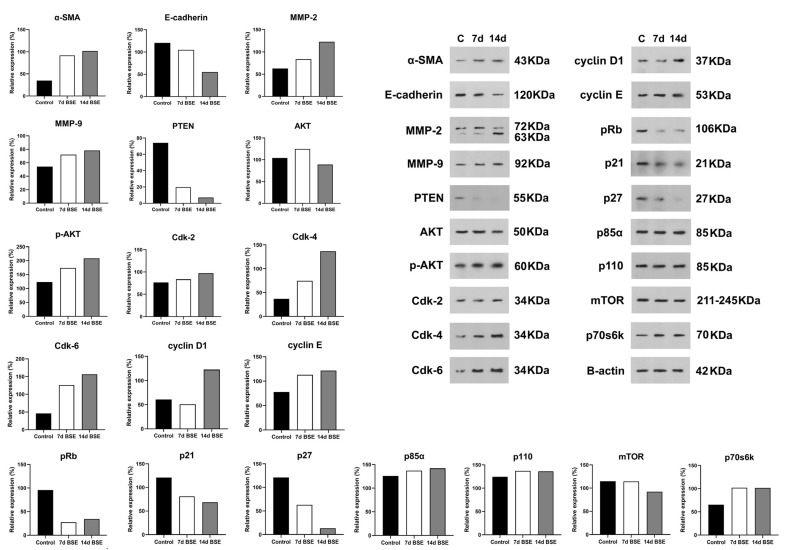
The protein expression of proliferative proteins after treating of HFF cells with 7 d and 14 d extracts of *Ophiocoma cynthiae*. The protein bonds of control group and the 7 d and 14 d extracts of brittle star can be observed on the Western blot gel. C, control.

Based on the MTT and in vitro migration assay results, the 7 d and 14 d BSE were chosen for Western blot analysis. After treating HFF cells with 7 d and 14 d BSE, the expression levels of α-SMA, MMP-2, MMP-9, p-AKT, Cdk-2, Cdk-4, Cdk-6, cyclin E, p85α, p110, and p70s6k increased (Figure 6 and Figure S8). However, the expression levels of E-cadherin, PTEN, pRb, p21, p27, and mTOR decreased after treating HFF cells with 7 d and 14 d BSE (Figure 6). Moreover, the expression of AKT increased and decreased following treatment with the 7 d and 14 d BSE, respectively. However, the cyclin D1 expression showed a decrease and an increase in expression after cell treatment with 7 d and 14 d BSE, respectively (Figure 6).

### 2.7. Three Bioactive Molecules in O. cynthiae Extracts Had the Highest Affinity to Apoptotic Peptides

According to the available studies, 14 out of the 36 identified compounds were selected as promising compounds in wound healing, and their binding affinity to 20 proteins was studied. Molecular docking was performed using Autodock Vina, and 10 conformations were obtained. The conformations with smaller values of binding affinity, ΔG [U total in kcal/mol], were selected as the best ones. Table 2 shows the binding affinities related to these conformations.

**Figure 7:**
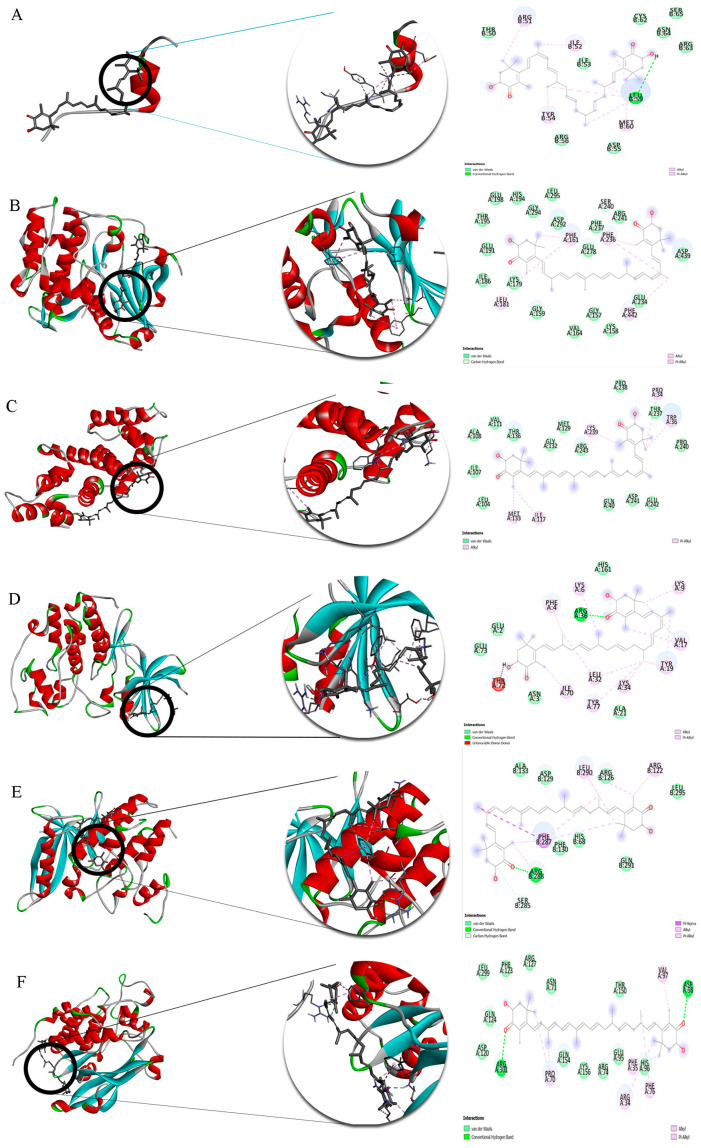

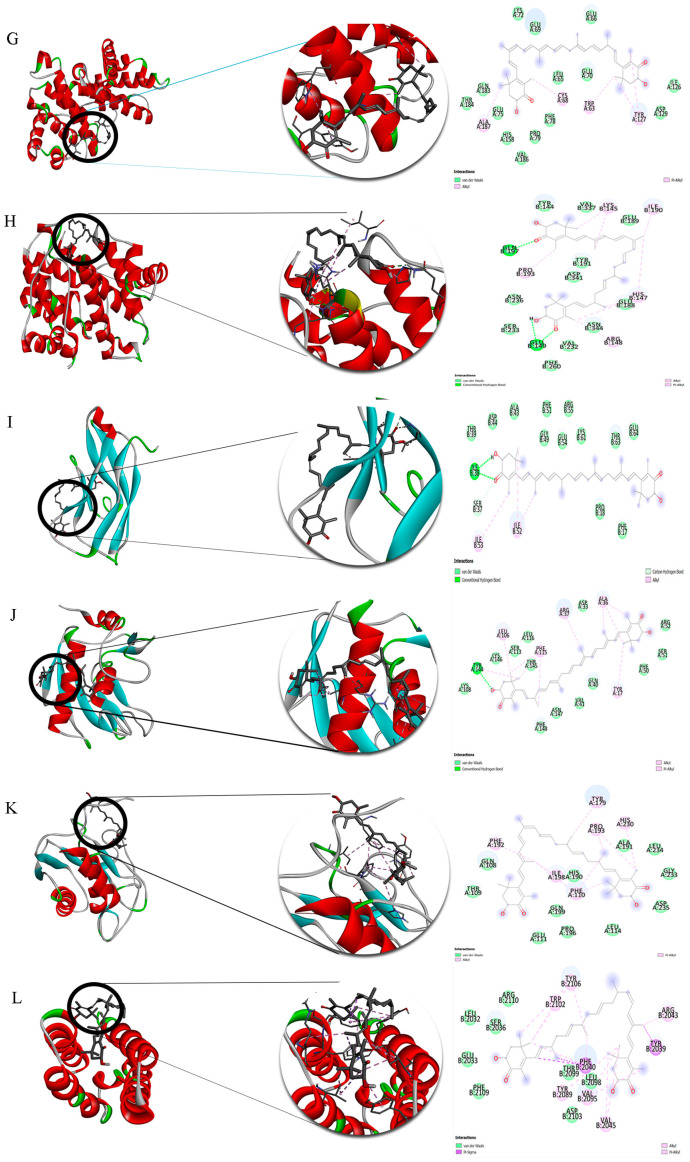

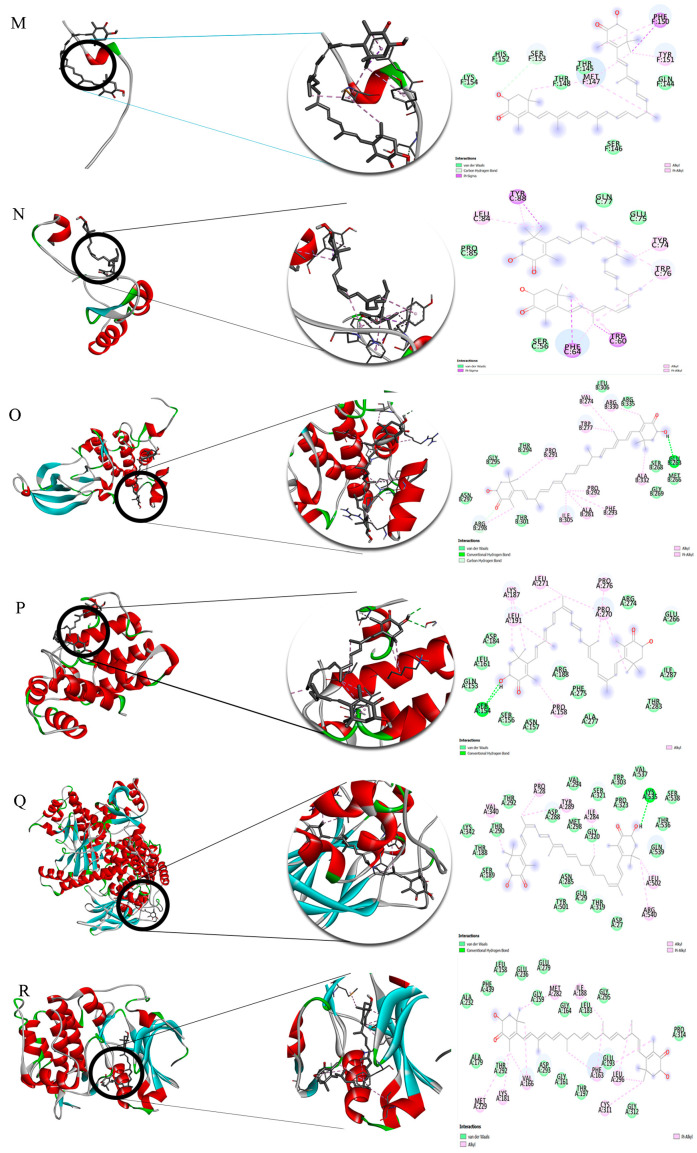

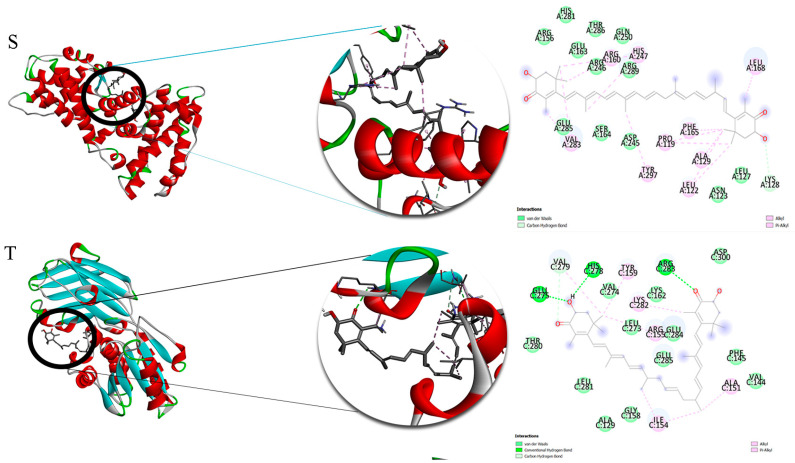
The 3D plot of the binding sites and the 2D plot of interactions of astaxanthin with (**A**) 4EBP1, (**B**) AkT, (**C**) α-SMA, (**D**) Cdk2, (**E**) Cdk4, (**F**) Cdk6, (**G**) Cyclin D1, (**H**) Cyclin E, (**I**) E-cadherin, (**J**) MMP2, (**K**) MMP9, (**L**) mTOR, (**M**) p21, (**N**) p27, (**O**) p70S6K, (**P**) p85α, (**Q**) p110, (**R**) pAkT, (**S**) pRb, and (**T**) PTEN.

Table 3 indicates binding affinities in the range of −2.8 to −10.6 kcal/mol. The lowest binding affinities are those related to the interaction between ligands with p21 and 4EBP1. Among the selected compounds, astaxanthin shows a better affinity for all targets. The intermolecular interactions of these compounds with targets are shown in Figure 7A–T.

**Figure 8:**
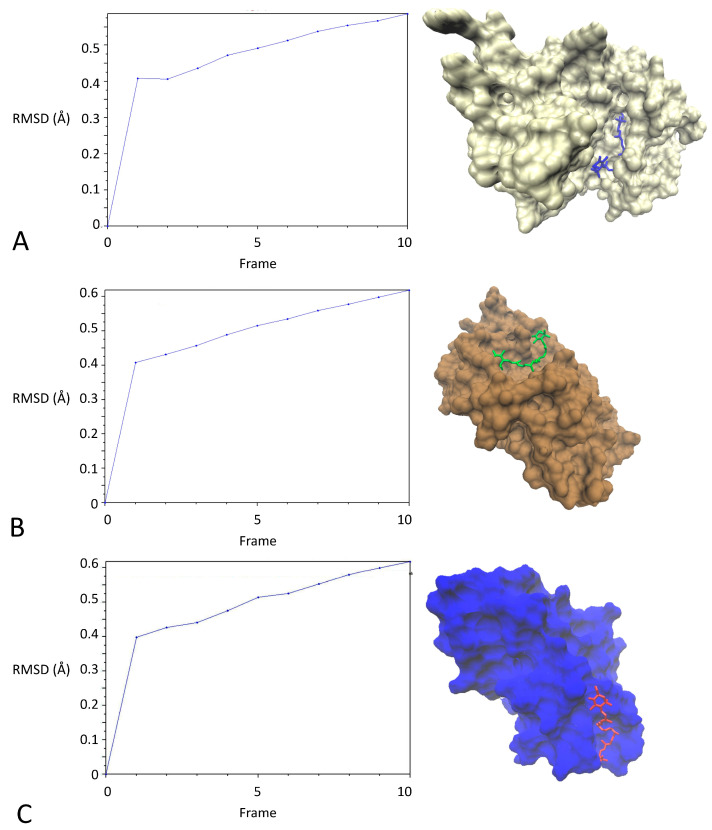
3D structures and RMSDs of complexes of Astaxanthin with (**A**) pAkT, (**B**) PTEN, and (**C**) Cdk6.

Then, Astaxanthin, which showed the highest affinity for the targets, was subjected to molecular dynamics studies. 3D structures of Astaxanthin complexes with the three targets for which it showed the highest affinity (pAkT, PTEN, and Cdk6), along with their RMSD values, obtained on the basis of molecular dynamics simulations, are shown in Figure 8. The obtained RMSDs demonstrate the stability of citrinin complexes during 10 runs of molecular dynamics simulation. Lower values of RMSD indicate protein complexes with greater stability. The RMSD values were 451, 0.461, and 0.464 Å for interaction of Astaxanthin with pAkT, PTEN, and Cdk6, respectively. It can be seen that the complex of Astaxanthin with pAkT, with the lowest RMSD value, is more stable than the other ones. This finding is in line with those predicted through docking simulations.

### 2.8. In Vivo Wound Healing Model Evaluation

**Figure 9:**
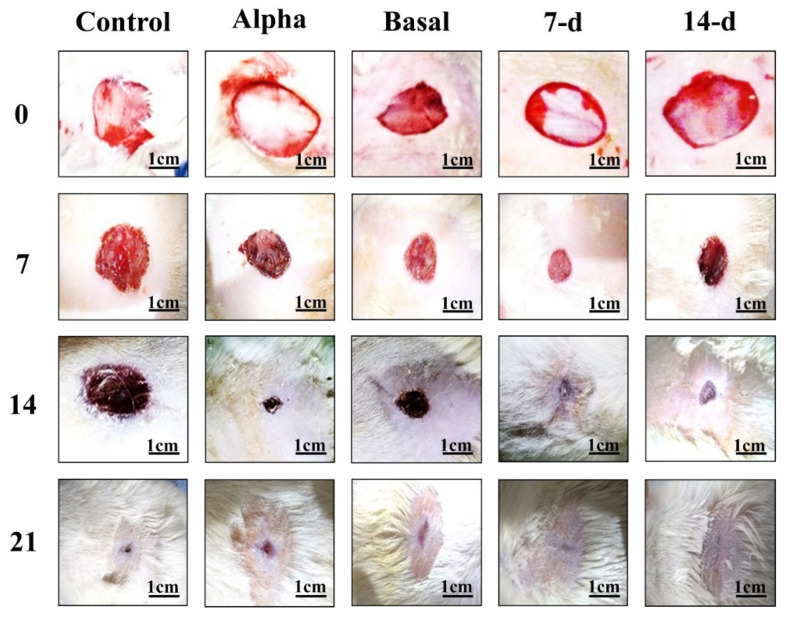
Wound area of different treatments in in vivo study of mouse wound healing model. Scale bar: 1 cm.

**Figure 10:**
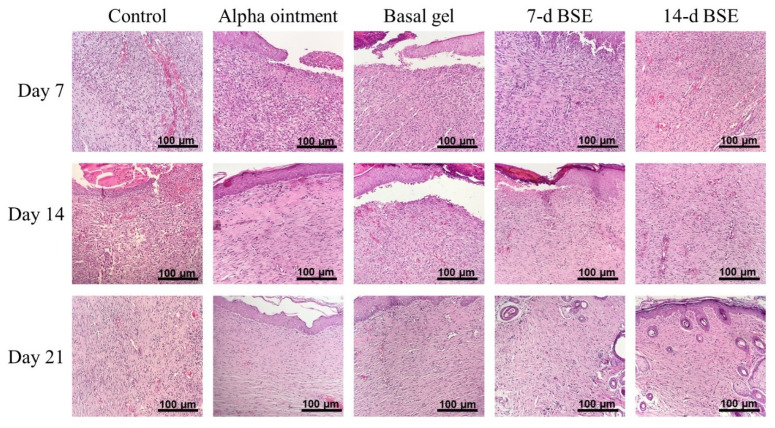
Histopathological evaluation of rat skins 7, 14 and 21 days after wound induction. Each group was assessed at 200× magnification. The scale bar is 100 µm.

The wound healing process of all 45 rats was measured macroscopically (Figure 9). The wound areas in the 7 and 14 d BSE gel groups on day 7 were close to half of their original size; however, the 7 d BSE gel seemed to have better results. Treatment with the Alpha ointment did not close the wound as well by day 7 as was observed for the 7 and 14 d BSE gels. Moreover, the wounds seemed to have closed by day 14 when treated with the 7 and 14 d BSE gels or the Alpha ointment. However, unlike the Alpha ointment, no scar formed for the wounds treated with the 7 and 14 d BSE gels. At this time, the wounds receiving the basal gel and control treatments were still open. On day 21, all wounds were closed, and scar formation was observed in the control, Alpha ointment and basal gel groups, but wasn’t observed in the 7 and 14 d BSE gel groups. The histological qualitative evaluation of the rat wounds revealed that, on days 7, 14 and 21, fibroblast maturation and collagen formation were mild in the Alpha ointment and basal gel groups, and were moderate in the 7 and 14 d BSE gel groups (Figure 10).

**Figure 11:**
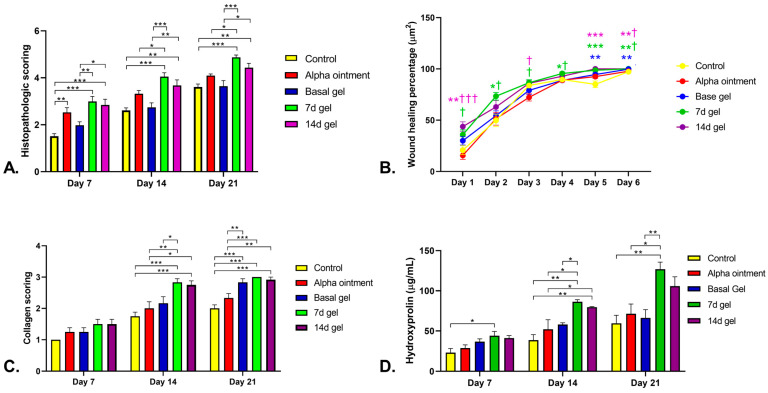
In vivo histopathological statistical evaluation of brittle star. (**A**) Histopathological scoring at 7, 14 and 21 days after wound induction in an in vivo wound healing model. (**B**) Wound healing percentage analysis of different treatments in in vivo study. * *p* < 0.05, ** *p* < 0.01 and *** *p* < 0.001 shows differences of 7d or 4d gels with control in a same day; ^†^ *p* < 0.05, and ^†††^ *p* < 0.001 shows differences of 7d or 4d gels with alpha ointment in a same day. (**C**) Collagen scoring analysis of different treatments in in vivo study. (**D**) Hydroxyproline measurement of wound sample of different treatments in in vivo study. * *p* < 0.05, ** *p* < 0.01 and *** *p* < 0.001.

Histopathological scoring of the wound healing of skin excisions was performed on days 7, 14 and 21 for all groups (Figure 11). Specifically, on day 7, the histopathological scores of the wounds treated with 7 d BSE gel were higher than those of wounds receiving the control or basal gel treatments (*p* < 0.001 and *p* = 0.007, respectively, Figure 11A). Additionally, the histopathological scores of the wounds treated with the 14 d BSE gel were higher than those of wounds receiving the control or basal gel treatments, too (*p* < 0.001 and *p* = 0.025, respectively, Figure 11A). Moreover, this score for the Alpha ointment was higher than that of the control group (*p* = 0.006, Figure 11A). On the 14th day, the histopathological scores of the wounds treated with the 7 d BSE gel were higher than those of wounds receiving the control and basal gel treatments (*p* < 0.001, Figure 11A). In contrast with day 7, on day 14, the histopathological scores of wounds treated with the 7 d BSE gel were higher than that of wounds treated with Alpha ointment (*p* = 0.043, Figure 11A). Furthermore, similar to on day 7, on day 14, the histopathological scores of wounds treated with the 14 d BSE gel were higher than those of wounds receiving the control and basal gel treatments (*p* < 0.01, Figure 11A). In contrast with day 7, there was no difference between the control and Alpha ointment groups (Figure 11A). Additionally, on day 21, the histopathological scores of wounds treated wtih the 7 d BSE gel were higher than those of the groups receiving the control, Alpha ointment and basal gel treatments, similar to day 14 (*p* < 0.001, *p* = 0.013 and *p* < 0.001, respectively, Figure 11A). Furthermore, on day 21, the wounds’ histopathological scoring of the 14 d gel was higher than control and basal gel, similar to days 7 and 14 (*p* < 0.008 and *p* = 0.012, respectively, Figure 11A).

Another parameter that was evaluated was wound healing percentage. On day 1, the wound healing percentage of the 14 d BSE gel treatment was higher than that of the control group (*p* < 0.004, Figure 11B). Furthermore, the wound healing percentage of 7 and 14 d BSE gel treatments was higher than that of the Alpha ointment (*p* = 0.013 and *p* < 0.001, respectively, Figure 11B). In contrast with day 1, on day 2, the wound healing percentage of the 7 d BSE gel treatment was higher than that of the control group (*p* = 0.029, Figure 11B), and that of the 14 d BSE gel treatment was not different from those of the control group and Alpha ointment (*p* > 0.05). Additionally, the wound healing percentage of the 7 d BSE gel treatment was higher than that of the Alpha ointment (*p* = 0.049, Figure 11B). On both days 1 and 2, there was no difference between the 7 and 14 d BSE gel treatments and the basal gel treatment. In contrast with days 1 and 2, on day 3, there was no difference between the 7 and 14 d BSE gel treatments and the control group treatment (*p* > 0.05). The wound healing percentage of the 7 and 14 d BSE gel treatments was higher than that of the Alpha ointment treatment on day 3 (*p* < 0.05, Figure 11B). On the fourth day, the wound healing percentage of the 7 d BSE gel treatment was higher than those of the Alpha ointment and the basal gel treatment (*p* < 0.05, Figure 11B). Further results on day 5 showed that the healing percentages of the basal gel and 7 and 14 d BSE gels treatments were higher than that of the control group treatment (*p* = 0.007, *p* < 0.001 and *p* < 0.001, respectively, Figure 11B). Similar to day 5, on day 6, the wound healing percentages of the basal gel and the 7 and 14 d BSE gels treatments were higher than that of the control group treatment (*p* < 0.01, Figure 11B). Moreover, the wound healing percentages of the 7 and 14 d BSE gel treatments were higher than that of the Alpha ointment (*p* < 0.05, Figure 11B).

The collagen formation scoring was also evaluated in more detail (Figure 11C). The results demonstrated that there was no difference between the control, the Alpha ointment, the basal, and the 7 and 14 d BSE gel groups with respect to collagen score during the first week of analysis (*p* > 0.05, Figure 11C). In contrast, on day 14, the collagen scores of the 7 and 14 d BSE gels were higher than that of the control group (*p* < 0.001, Figure 11C). In addition, the collagen scores of both 7 and 14 d BSE gel were higher than that of the Alpha ointment (*p* = 0.006 and *p* = 0.017, Figure 11C). In contrast to the 14 d BSE gel, the collagen score of the 7 d BSE gel was higher than that of the basal gel (*p* = 0.044, Figure 11C). At day 21, the collagen scores of the basal gel and the 7 and 14 d BSE gels were higher than that of the control group (*p* < 0.001, Figure 11C). In line with the 14-day collagen scoring, the collagen scores of the 7 and 14 d BSE gels were higher than that of the Alpha ointment (*p* < 0.001 and *p* = 0.002, respectively, Figure 11C) at 21 days. Furthermore, the collagen score of basal gel at 21 days was higher than that of the Alpha ointment (*p* = 0.009, Figure 11C).

### 2.9. Hydroxyproline Measurement

The results of hydroxyproline measurement demonstrated that, on day 7, only the 7 d BSE gel had a higher value than the control group (*p* = 0.034, Figure 11D). Furthermore, on day 14, the hydroxyproline levels in both the 7 and 14 d BSE gels treatment were higher than those in the control group (*p* = 0.001 and *p* = 0.003, respectively, Figure 11D). In addition, both of the previous treatments had higher hydroxyproline levels than the Alpha ointment (*p* = 0.012 and *p* = 0.049, respectively, Figure 11D). Moreover, in contrast with the 14 d BSE gel treatment, the hydroxyproline level in the 7 d BSE gel treatment was higher than that in the basal gel treatment (*p* = 0.043, Figure 11D). Further analysis on day 21 demonstrated that the 7 d BSE gel treatment had a higher hydroxyproline level than the control group, Alpha ointment and basal gel (*p* = 0.003, *p* = 0.016 and *p* = 0.008, respectively, Figure 11D).

## 3. Discussion

In the present study, we evaluated the arm regeneration of the Persian Gulf brittle star *O. cynthiae*, the compounds isolated from it using hydroalcoholic extract, and their effect on in vitro and in vivo wound healing models. *O. cynthiae* has the potential to undergo regeneration of an amputated arm. On the basis of primary observation and a pilot study performed in preparation for the current study, *O. cynthiae* completes the regeneration of an amputated arm in an average time of 14 days. The process of arm regeneration among different types of brittle star remains almost the same [17], although some parts of the process, for example the time of regeneration, differ between species [17]. A previous study on brittle star arm regeneration showed that the total time required for the regeneration process in *O. longicaudum* and *A. wliformis* was 12 weeks and 24 days, respectively [17]. However, in line with the results of the present study, *Ophiocoma* sp. were shown to be able to achieve a higher total regeneration than other species, such as ophiodermatid species, therefore causing it to regenerate faster [17]. The process of arm regeneration begins with the immediate protection of the wound site by means of arm shields and muscle contraction upon arm amputation. This process is undertaken in order to close the coelomic canals and cavities of the brittle star to avoid the loss of coelomic fluid. Additionally, two buds are symmetrically formed in the upper part of the wound. These buds form the new spines of the newly regenerating arm. Subsequently, early on day 2, the process of re-epithelialization commences, covering the wound area. After the completion of re-epithelialization, early on days 6 and 7, a new bud containing blastema begins to form. This new bud proliferates, ultimately forming the radial nerve cord and the radial water canal. The re-growth of the new arm is completed on day 14. In line with the present study, the arm regeneration process for different brittle stars has been shown in previous studies to be almost the same as that described in the current study, although more details of the regeneration process have been described in previous studies [16,17].

In order to determine the molecules produced during the arm regeneration of brittle stars and their effects on the regeneration process, GC-MS analysis was performed. GC-MS analysis showed that the 0 h BSE mostly had antmicrobial and anti-inflammatory effects. This indicates that in the early hours following arm amputation, the brittle star produces some antibacterial and antifungal materials in order to prevent putrefaction of the wound area. In line with the present study, it has been demonstrated in another study that the crude extract of the brittle star possesses antimicrobial activity [79]. Moreover, the 0 h BSE exhibited antioxidant activity. As starfish metabolism increased during arm regeneration [80] and there was an increase in the number of free oxidative radicals, antioxidant production, especially in the early days of regeneration, seems to be vital. Another study of the brittle star with results in line with the current study showed that the crude extract of the brittle star demonstrated antioxidant activity [22]. In addition, the 0 h BSE had other effects, such as muscle contraction and anti-proliferation effects. The muscle contraction seems to be vital for wound protection. Because the wound is open after arm amputation, the brittle star covers the wound area and coelomic cavity with shields at the tip of the arms, along with muscle contraction [17]. The desulphosinigrin compound of 0 h BSE has been shown to have antimicrobial and antiproliferative effects [35,36]. This antiproliferative effect seems to be vital to suppressing the overgrowth of microbes, which live symbiotically around the body of the brittle star. Moreover, the brittle star crude extract and some bioactive substances isolated from the brittle star, such as saponins, have been shown to have anti-proliferation and anti-metastatic properties [22]. However, a previous study showed that the cholesterol compound detected in this extract has a cell proliferative effect [31]. As mentioned previously, in the early hours of day 1, two regeneration buds started to form to produce new spines. Based on morphological and chemical analysis, it can be concluded that cholesterol is a key element for triggering proliferation and bud formation.

In addition, anti-inflammatory substances were also produced by brittle stars on day 0, preventing inflammation during the arm regeneration process. A previous study reporting results that were in line with those in the current study showed that the saponin fraction of BSE possesses an anti-inflammatory effect [26]. Additionally, the neuroregeneration process was triggered at day 0 of regeneration through the excretion of a Pseduosarsasapogenin-5,20-dien methyl ether compound. This indicated that the process of neuroregeneration started during the early hours following arm amputation. Moreover, the same compound was produced on day 3 of regeneration, continuing the neuroregeneration process. In line with the results of the present study, a previous report on brittle star arm regeneration showed that coelomic and neural tube regeneration started with blastema formation on days 3–4 [17]. While these studies of brittle star regeneration evaluated the regeneration process on a physical basis [16,17], to the best of our knowledge, there are no studies evaluating the arm regeneration process of brittle star on a chemical basis. Additionally, as mentioned before, bud formation and neuroregeneration take place faster in *O. erinaceus* than in *A. wliformis* [17].

The compounds constituting the brittle star extract at different regeneration times were evaluated. To the best of our knowledge, this is the first study to evaluate the compounds produced during brittle star arm regeneration. The presence of antimicrobial and anti-inflammatory compounds was observed in all BSEs, regardless of time. This shows that from the moment the wounded area was closed until the end of the regeneration process, there was to be no presence of contamination, microbes, or inflammation, which could probably interfere with the regeneration process. There were only three compounds, that were produced within 3 h of arm amputation. One compound in the 3 h BSE had an anti-proliferation effect. The small number of compounds present at 3 h that were not present at 0 h shows that most of the compounds required for the regeneration process are excreted by the brittle star almost immediately following amputation. The 3 d BSE had the same compounds as the 0 h brittle star compounds. The main effects of the compounds in the 3 d BSE were antimicrobial and anti-inflammatory, which is the same as the the compounds in the 0 h BSE.

The main effects exerted by the compounds in the 5 d BSE were antimicrobial and anti-inflammatory, the same as in the previous extracts. Moreover, two compounds with antioxidant effects were found in the 5 d extract that were able to accelerate the proliferation and wound healing process [81]. In addition antimicrobial and anti-inflammatory compounds, there are other compounds that trigger other proliferation processes. The Ethyl.alpha-d-glucopyranoside compound, which was detected in the 7 d BSE, was shown in a previous study to have cell proliferation and ECM production effects [33]. Moreover, cholesterol was also present in this extract, which also induced the proliferation process [31]. Both of these compounds triggered proliferation, leading to the formation of another bud—a bud containing blastema in the oral part of the arm for the production of a new coelomic and nerve canal, as well as, more generally, a new arm. In addition, two new antioxidant compounds—astaxanthin and hexadecanoic acid, 2-hydroxy-1-(hydroxymethyl)ethyl ester—were detected in the 7 d extract that could be vital for this stage of regeneration due to the increased proliferation and cell metabolism [80]. Moreover, the GC-MS analysis of 14 d BSE showed that at this stage of regeneration, cell migration was induced by octadecanoic acid, methyl ester (Methyl stearate) compounds [34]. Furthermore, another compound was excreted, referred to as 1,2-Benzenedicarboxylic acid, bis(2-methylpropyl) ester, which in a previous study was shown to possess an anti-proliferation effect [70]. Additionally, studies have shown that this compound and the hexadecanoic acid, methyl ester compound have antifibrotic effects [70,75]. These compounds, which also have an antifibrotic effect, prevent scar formation, and taken together lead to the formation of an arm similar to that which was amputated. The results also showed that on day 14 of brittle star arm regeneration, the proliferation process stops, in order to prevent overproliferation of cells and tissue production.

Based on the pilot in vitro study, as well as on the cell proliferation and migration effects of the compunds found in the 7 and 14 d brittle star extracts, the 7 and 14 d BSEs were chosen for use in our in vitro study. Firstly, the MTT assay was performed for the cell proliferation assay of extracts. Although the 7 d BSE contained cell proliferative compounds, the MTT analysis showed that there was no difference between the group treated with the 7 d extract and the control group. However, there were higher proliferation effects in the 14 d BSE-treated group than in the 7 d BSE group. However, there were no significant differences between the 14 d BSE treatment group and the control group.

As the MTT assay of the BSEs showed that the BSEs did not have a toxic effect on HFFs, an in vitro study on HFFs was performed. The in vitro model of wound healing in cell migration assay demonstrated that 7 and 14 d BSEs had a greater effect on the wound healing on HFFs compared to the control group within 24 h. Furthermore, there was a significant difference between the 7 and 14 d extracts and other BSEs. Due to the significant difference between the 7 and 14 d BSEs with respect to in vitro wound healing, it is logical that there are compound(s) that, to some extent, cause these proliferation effects. On the basis of the GC-MS analysis, the compound Ethyl-α-ᴅ-glucopyranoside was identified, the effect of which on fibroblast proliferation and ECM production has previously been reported [33]. This compound was only observed on the basis of the GC-MS analysis of the 7 d BSE, and its presence was not observed in the other BSEs—not even in the 14 d extract. Moreover, cholesterol was found in the 7 d BSE that induced cell proliferation [31]. Furthermore, the benzenepropanoic acid, 3,5-bis(1,1-dimethylethyl)-4-hydroxy-, methyl ester compound in the 14 d BSE has been reported previously, along with its antioxidant activity [76]. Additionally, another compound in this extract, octadecanoic acid, methyl ester (Methyl stearate), was shown to induce cell migration [34]. These compounds could be the reason for the different proliferative activities of 7 and 14 d BSEs in comparison with other BSEs and the control group.

In the current study, the expressions of the proliferative and antiproliferative proteins were examined. It has been shown that the p13K/AKT pathway is related to cell proliferation, migration and survival [82]. Increased AKT in the p13K/AKT pathway is correlated with an increase in cell proliferation and survival [83]. It has also been shown that the PI3K/Akt/mTOR/p70S6K pathway plays a major role in cell proliferation [84]. The MMP-2 and MMP-9 regulations have been shown to be mediated by the p13K/AKT pathway, and these MMPs are involved in cell migration [85]. In the current study, there was an increase in these proteins, which are involved in cell proliferation and migration, after cell treatment with 7 d and 14 d BSEs. However, there was a decrease in antiproliferative protein expression following cell treatment. E-cadherin plays a key role in the suppression of tumor cell metastasis and invasion, and exerts tumor suppressor effects [86]. Previous studies have shown that PTEN and p21 also play key roles in tumor suppression [87,88]. The p21, p27, and p57 proteins are a group of proteins that inhibit all types of CDKs. This leads to the inhibition of cell cycle progression, and they have antiproliferative effects [89]. Moreover, it was shown that the downregulation of p27 and pRb expression has synergic effects on cell proliferation, downregulating it [90].

It is clear that the cell cycle has four stages, and every stage is regulated by specific checkpoints. Some of these regulatory agents include cyclins and CDKs [91,92]. The cyclin D1-CDK4/CDK6 complex was shown to interfere with the cell cycle from the G0 to the G1 phase, and the entry of the cell into the G1 phase depends on this pathway [92]. However, it has been reported that the cyclin E/CDK2 complex plays a major role in cell entry into the S phase from the G1 phase [92,93]. In the current study, the expression of CDK-4 and CDK-6 increased with both treatments, while the protein expression decreased with cyclin D1 and increased with the 7 d and 14 d BSE treatments, respectively. These results demonstrate the possible effects of 7 d and 14 d BSE on the entry of cells into the G1 phase from G0. Moreover, expression of cyclin E and CDK2 increased after treatment. However, the protein expression of the 14 d BSE was higher than that of the 7 d BSE. These results demonstrate that treatment with both extracts may effect the entry of cells into the S phase from G1. Taken together, it seems that the 7 d and 14 d extracts accelerate cell proliferation by increasing the expression of proliferative proteins and decreasing the expression of anti-proliferation proteins.

Before in vivo study, computational modeling was performed. In this study, lower values of binding affinity, ΔG [U total in kcal/mol], indicate more favorable ligand–protein interaction, as their complex is more stable. Considering docking scores lower than −4 to denote a good interactive relationship between the component and the protein, nearly all of the selected compounds can be observed to exhibit a good affinity for most of the targets. However, astaxanthin showed a better affinity for the targets. Accordingly, it can be concluded that this is the most affective component for which its binding with the target can be controlled using different intermolecular interactions, including van der Waals interactions, hydrophobic interactions and hydrogen bonds (Figure 7). In general, all of these compounds are able to effectively interact with the investigated proteins and affect the treatment process.

As a result of the better cell migration, MTT cell proliferation and Western blot analysis results of 7 and 14 d BSEs, we performed an in vivo study of 7 and 14 d BSEs. In this study, five treatment groups were defined for the rat wound healing model: the negative control group, without treatment; Alpha ointment, as a commercial positive control group; basal gel as another positive control group; and 7 and 14 d BSEs (BSE gels) as the two treatment groups. The Alpha ointment is a routine topical medication that is used worldwide for burn wounds [94]. Meanwhile, basal gel was included in order to determine its effect on the wound healing process, as well as to ensure that the effects of the 7 and 14 d BSE gels were not because of their basal gel. The 7 and 14 d BSE gels were observed to have better wound healing effects both macroscopically and histologically. Moreover, unlike the other groups of wound models, scar formation was not observed when using the 7 and 14 d BSE gels. The total results in the rat wound healing model showed that the 7 and 14 d BSE gels had a better effect than the control group with respect to wound healing. Furthermore, there were no significant differences between the basal gel and the control group, which means that the healing effect of the 7 d and 14 d BSE gels was not related to their basal gel. In addition, the wound healing effects of the 7 d BSE gel were higher than those of the 14 d BSE gel, but the difference was not significant. Moreover, the Alpha ointment, as a common treatment for the treatment of burn wounds, exhibited good healing effects on the rat wound healing model, demonstrating a higher rate of wound healing than the control group. Meanwhile, the 7 d BSE gel, unlike the 14 d BSE gel, had a greater effect on wound healing than the Alpha ointment after week 2, demonstrating that the 7 d BSE gel had a better yield than the Alpha ointment or the 14 d BSE gel during the wound healing process. In addition to the good wound healing effects of the 7 and 14 d BSE gels, no scar formation was observed in these two groups, as mentioned previously. This effect could be due to the presence of compounds such as 1,2-Benzenedicarboxylic acid, bis(2-methylpropyl) ester and hexadecanoic acid, and methyl ester, which possess antifibroitc effects [70,75].

The collagen scoring demonstrated there were no differences between any of the treatment groups during the first weeks of treatment. During the second week of treatment, there was a larger significant difference between 7 and 14 d BSE gels than between the normal and Alpha ointment treatment groups. During the third week of treatment, the same results were obtained. Moreover, the 7 d BSE gel, unlike the 14 d BSE gel, had a higher score of collagens than the basal gel, indicating that the 7 d BSE gel possessed a better yield. While this was the case for the second week, this difference was not observed in the third week of treatment. Hydroxyproline, which is a major subunit of collagen polypeptide and plays a key role in collagen stability, was also measured [95]. It is also a good marker for collagen production and degradation rate [95]. The hydroxyproline measurements also implied the same collagen scoring, with slight differences. In the first week, only the 7 d BSE gel had a higher value than the control group. Moreover, in the third week of treatment, only 7 d BSE gel had a hydroxyproline value that was higher than that of the other groups, as opposed to collagen scoring, the 14 d BSE gel also demonstrated its difference from the other groups. Taken together, both the collagen and hydroxyproline measurements showed that the 7 d BSE gel had the best effect on wound healing ability in a rat wound healing model—even better than the 14 d BSE gel. Despite being in second place, the 14 d BSE gel was shown to have a good regeneration effect in a rat wound healing model.

## 4. Materials and Methods

### 4.1. Ethical Approval Statements

This investigation was performed in accordance with relevant guidelines and regulations of animal studies, and all experimental protocols were approved by the ethical committee of Bushehr University of Medical Sciences (Permission number: IR.BPUMS.REC.1399.128) and Shiraz University of Medical Sciences (Permission numbers: IR.SUMS.MED.REC.1399.362 and IR.SUMS.MED.REC.1399.314).

### 4.2. Brittle Star Collection and Adaptation

Brittle stars, *O. cynthiae*, were collected from the shores of Nayband gulf, Asaluyeh, Bushehr. Adult *O. cynthiae* ranging in overall diameter between 15 and 25 cm were selected. They were transferred and cultured in an aquarium containing aerated artificial sea water in the Center of Marine Comparative and Experimental Medicine, Bushehr University of Medical Sciences, Bushehr, Iran. The light cycle was 12 h light and 12 h dark, water temperature was 25 °C, and the brittle stars were fed once a day with salt water aquaculture food (Biomar^®^, Aarhus, Denmark). They were adopted after 1 week.

### 4.3. Brittle Star Species Identification

The brittle star samples were examined under a stereomicroscope (Cobra Micro Zoom, MZ1000, Micros Co., Tratschweg, Austria). Species identification was carried out based on adult specimens using the key indexes described below, on the basis of previous studies [28,29,30,96,97,98,99,100]. the form of the disc, the number of granules of millimeter length on the disc, the extension of oral surface granules of the disc, the number and shape of podial (tentacle) scales, the shape of the oral shield, the arm spines and aboral arm plates, and the coloration of feet tube were examined.

### 4.4. Macroscopic and Histological Evaluation of Amputated Arm Regeneration of Brittle Stars

The wound healing of the brittle stars was analyzed on the basis of macroscopic and microscopic imaging at different times during the formation of the new limb. Macroscopic images were captured using a stereo microscope (Cobra Micro Zoom, MZ1000, Micros, Sankt Veit an der Glan, Austria), phone camera (Samsung S8 plus, Seoul, Republic of Korea) and microscope-phone adaptor (PerciaVista Co., Shiraz, Iran). Based on the information regarding morphological changes during arm regeneration in *O. erinacenus* obtained in the present study and a modification of sampling times described the previous studies [19,101], six times were chosen at which to perform histological analysis. Firstly, brittle stars were cold anesthetized on ice. One-third of brittle stars’ arm tips were cut with sterile scalpel blades, and brittle stars were transferred back into the aquarium. The amputated arms were sampled at 0 h, 3 h, 3 d, 5 d, 7 d, and 14 d after amputation. Tissue samples were fixed in 4% formaldehyde in 1 M NaCl solution for one week. Then, tissues were decalcified using 3% nitric oxide for 24 h. The fixed and decalcified tissues were processed using a tissue processor (Didsabz, Tehran, Iran) as follows: 70% ethanol for 90 min, 80% ethanol for 45 min, first stage of 96% ethanol for 90 min, second stage of 96% ethanol for 60 min, first stage of absolute ethanol for 75 min, second stage of absolute ethanol for 100 min (all alcohols were purchased Zakaria Jahrom Co., Shiraz, Iran), two stages of xylene (Carlo Erba Reagents Co., Val-de-Reuil, France) for 100 min each stage, and two wax stages at 58 °C for 75 min and 45 min, respectively. Afterward, the tissues were placed into wax embedding blocks and stored at room temperature.

Paraffin-embedded tissues were cut to a thickness of 10 µm and placed onto coated glass using albumen glue (*v*/*v* white egg/glycerol). Slices were dewaxed and rehydrated in xylene and 100%, 95%, 80%, and 75% ethanol solution, respectively (each stage was performed 2 times and for 5 min). Sections were stained with hematoxylin (Arian Cellul Sepehr, Tehran, Iran) for about 10 min and then rinsed with tap water for 5 min. Subsequently, slides were placed into an acid–alcohol solution containing 1% hydrochloric acid (Merck, Darmstadt, Germany) in 70% ethanol for 3–6 s until the slices turned red. Then, the slides were rinsed with tap water for about 5 min. Next, eosin dye (Arian Cellul Sepehr, Tehran, Iran) was used for 15 min, and the slides were rinsed with distilled water for 5 min. For dehydration and clearing, sections were placed 2 times in 70%, 96%, and 100% ethanol solutions and xylene stages for 5 min each time. Finally, 1 to 2 drops of Shandon™ Consul-Mount™ glue (Thermo Fisher Scientific Inc., Erlangen, Germany) were dropped onto the dry slice, before quickly covering them with a clean coverslip. Under a light microscope (Olympus, Tokyo, Japan), the nucleus was blue and the cytoplasm and fibrous tissue were in shades of red.

### 4.5. Brittle Star Extractions

The brittle stars were kept in the aquarium for 0 h, 3 h, 3 d, 5 d, 7 d, and 14 d after amputation. Forty grams of the intact body of the brittle stars at different times following arm amputation was chopped off and extracted with 200 mL of 70% ethanol under stirring for five days at room temperature. The extracted solution was filtered using 100 µm Whatman^®^ filter paper (Sigma-Aldrich Co., WHA1440125, Darmstadt, Germany) and concentrated with a vacuum rotary evaporator (Laborota 4003-control, Heidolph Instruments GmbH & CO. KG, Schwabach, Germany). The extracts were kept at −80 °C until further use.

### 4.6. Gas Chromatography–Mass Spectrometry (GC-MS) Analysis

The brittle star hydroalcoholic extract was subjected to the 7890B Agilent Gas Chromatography–Mass Spectroscopy (GC-MS, Agilent Technologies, Santa Clara, CA, USA). Electron ionization (EI) mass spectra (scan range, *m*/*z* 50–500, Agilent19091s-443, Agilent Technologies, Santa Clara, CA, USA) were obtained using electrons with an energy of 70 eV, and a filament emission of 0.5 mA. The GC separations were carried out using an HP-5MS UI capillary column ((30 m × 0.25 mm × 0.25 µm) i.d., film thickness 0.5 µm)). Helium was used as the carrier gas (flow: 0.8 mL/min) for EI. The GC oven was programmed with an increase in temperature of 5 °C/min from 80 °C starting 3 min after sample injection, and then held at 250 °C for 10 min. The injection ports of the gas chromatograph, transfer line, and ion source of the 5977MSD were maintained at 240, 250, and 270 °C, respectively. The separated compounds were identified by matching them with the compound data from the National Institute of Standards and Technology (NIST MS database, 2014) library, and the relative (%) amount of each component was measured by comparing its average peak area to the total areas.

### 4.7. MTT Assay

HFFs were cultured in Dulbecco’s Modified Eagle Medium (DMEM, Gibco, Life Technologies Co., MD, USA) supplemented with 10% fetal bovine serum (FBS, Kiazist Co., Shiraz, Iran) and 1% penicillin–streptomycin (Pen-Strep, Gibco, Life Technologies Co., MD, USA). Cell proliferation and vitality were measured using the MTT assay kit (Sigma-Aldrich Co., Darmstadt, Germany). Specifically, 4 × 104 cells were seeded per well in a 96-well plate and incubated with DMEM supplemented with 10% FBS. After 24 h, the media was removed, and 7 d and 14 d BSEs were added at a concentration of 1% to the wells (at least 4 wells for each concentration) for treatment groups. The concentration of the extracts was 8.5 g/mL 7 d and 14 d BSEs in dimethyl sulfoxide (DMSO, Sigma-Aldrich Co., Darmstadt, Germany). Then, the solutions with 1% concentration were added separately to cell culture media (10 µL extract solution in 990 µL culture media). Additionally, DMEM supplemented with 10% FBS and 1% DMSO was added to the wells to act as the control group. The plates were incubated at 37 °C and 5% CO_2_ for 72 h. Subsequently, the media was removed, and cells were washed with PBS. Cells were incubated with 0.5 mg/mL MTT (3-(4,5-dimethylthiazol-2-yl)-2,5-diphenyltetrazolium bromide) for 4 h in an incubator until intracellular purple formazan crystals appeared. Finally, DMSO was added to each well and incubated at 37 °C for 20–30 min until the cells had lysed and the purple crystals dissolved. In the final stage, cell absorbance was measured at 570 nanometers using an ELISA plate reader machine (BioTek, Paramus, NJ, USA).

### 4.8. In Vitro Cell Migration Assay

HFFs were seeded at a density of 5 × 104 in a 24-well cell culture plate. After that, the confluency of the cells reached 90%, and the middle area of each well was crushed using a 100 µL pipette tip with a diameter of 500 µm across the diameter of each well. Subsequently, as described for the previous concentration, 0 h, 3 h, 3 d, 5 d, 7 d, and 14 d BSEs were prepared in DMSO at a concentration of 8.5 g/mL. Then, these solutions were added separately to the cell culture media at a concentration of 1%. Three wells were considered for each group of extracts. For the control group, DMSO was added to cell culture media at a concentration of 1% in 3 wells. Cells were incubated at 37 °C with 5% saturation of CO_2_ and 95% humidity for 2 days. Images of each well were captured using an inverted microscope (Labomed TCM400 microscope, Labomed Co., Los Angeles, USA) and a microscopic digital camera (Tucsen TCH-1.4CICE camera, Tucsen Photonics Co. Fuzhou, China) after incubation for 0 h, 24 h, and 48 h.

Image analysis was performed using ImageJ software (Fiji-ImageJ x 64, V1.52p, US National Institutes of Health, Bethesda, Maryland, USA). Specifically, imported images were converted to 8-bit images using the “Image type” option in the “Image” panel. Then, the scale of all images was set using the “Set scale” option in the “Analyze” panel. In order to analyze the wound area, the wound area was manually cropped, and then layer duplicated. For this purpose, the borders of the wounds were selected using the “Rectangle” tool in the main menu of the ImageJ software. Three images each of the 0 h, 3 h, 3 d, 5 d, 7 d, and 14 d groups of BSEs after incubation times of 0 h, 24 h and 48 h were analyzed. The “MRI wound healing” tool was used, and by means of the “Find edges” method as well as by adjusting the threshold and radius, the free space between cells was measured. In addition, the minimum and maximum distances between cells in each image were measured manually using the “Line selection” tool in the main menu of ImageJ software. Data for the free area were extracted using the “Measure” option in the “ROI manager” panel and saved in the Excel “SCV” format. The distance data were extracted using the “Measure” option in the “Analyze” panel.

### 4.9. Western Blot Analysis

Western blot analysis was performed based on previously described standard procedures with some modifications [102]. Based on the results of MTT proliferation and cell migration assays, the 7 d and 14 d BSEs were used for Western blot analysis. Specifically, after the HFF cells were treated with both extracts for 72 h, the cells were lysed with RIPA buffer (50 mM Tris–HCl (pH = 8.0), 0.4% Nonidet P-40, 120 mM NaCl, 1.5 mM MgCl_2_, 2 mM phenylmethylsulfonyl fluoride, 80 μg/mL leupeptin, 3 mM NaF, and 1 mM DTT) at 4 °C for 20 min. The remaining lysates were centrifuged using the following protocol: 12,000× *g* spinning for 20 min at 4 °C. The protein concentration was measured using a Bradford protein assay. Then, the proteins were transferred to a microporous polyvinylidene difluoride membrane (Millipore, Molsheim, France). Membranes were then incubated in 5% bovine serum albumin (BSA, Sigma-Aldrich, Burlington, USA) blocking buffer for 1 h at room temperature. Subsequently, the membranes were incubated separately with the corresponding primary antibodies overnight at 4 °C. Immunoblotting was performed with rabbit anti-Pan-Akt, anti-β-Actin (C4), α-SMA, anti-E-cadherin, anti-MMP-2, anti-MMP-9, anti-PTEN, anti-p-AKT, anti-CDK2, anti-Cdk4, anti-Cdk6, anti-cyclin D1, anti-cyclin E, anti-pRb, anti-p21, anti-p27, anti-p85α, anti-p110, anti-mTOR, and anti-p70s6k antibodies (1:200) (Cell Signaling Technology, Danvers, MA, USA). Subsequently, the membranes were washed 3 times (10 min each) with tween buffer before incubating with HRP-conjugated goat anti-mouse or rabbit secondary antibodies. Then, in order to remove excess antibodies, membranes were washed 4 times. Finally, HRP activities were detected using ECL Plus Chemiluminescence Reagent (Amersham, Chalfont, UK) according to the protocol supplied with the kit.

### 4.10. Computational Details

The structures of selected compounds were downloaded from the PubChem database (http://www.ncbi.nlm.nih.gov/pccompound, accessed on 1 December 2022) for further docking analysis. The three-dimensional (3D) structures of 20 targets were obtained from the Protein Data Bank (PDB, http://www.rcsb.org/, 1 December 2022) (α-SMA: 5a37, E-cadherin: 1o6s, MMP9: 4jij, MMP2: 3ayu, PTEN: 3awf, AkT: 3mv5, pAkT: 1o6l, Cdk2: 6q4d, Cdk4: 2w96, Cdk6: 1blx, cyclin D1: 2w96, cyclin E: 7kjs, pRb: 4ell, p21: 5e0u, p27: 1jsu, p85α: 6d85, p110: 7jiu, mTOR: 1aue, p70S6K: 3a60, and 4EBP1: 1wkw). The targets were prepared for docking by adding missing hydrogen, repairing the side chain, treating the termini, fixing the protonation state, and removing water molecules before docking. The grid box of these targets was generated using the Computed Atlas of Surface Topography of proteins (CASTp 3.0). A molecular docking experiment was performed using Autodock Vina 1.1.2 software to detect the possible binding modes of ligands. All docking conformations were obtained, and the best was selected according to their binding affinity score (i.e., lower negative energy and RMSD ≤ 2 Å).

Then, molecular dynamics simulations were performed for 100 ns for the three compounds with the highest affinity using the NAMD2 and VMD (1 December 2022, version 1.9.3, Beckman Institute, Urbana, IL, USA) to validate the docking process.

### 4.11. Preparation of Therapeutic Gels

Carboxymethyl cellulose (CMC, Sigma Aldrich Co., Darmstadt, Germany) gel was used to deliver therapeutic substances. Specifically, 10 mg of the BSEs were weighed and dissolved by 5 drops of DMSO using a vortex device. The resulting solution was then poured into 98 mL of distilled water and stirred using a magnetic stirrer. Then, 2 g of CMC powder was slowly added to the solution and stirred for 2 h, before eventually being stored at 4 °C. Three types of gel were prepared for three different treatment groups: (A) gel with BSE from 7 days after arm amputation (7 d BSE gel); (B) gel with BSE from 14 days after arm amputation (14 d BSE gel); and (C) gel without BSE but with 5 drops of DMSO.

### 4.12. Animal Model of Wound Healing

Forty-five male Sprague Dawley rats weighing an average of 220 ± 10 g and with an age of 3 months were purchased from the Center of Comparative and Experimental Medicine, Shiraz University of Medical Sciences. The rats were housed in separate cages for one week with an optimum temperature of 24 ± 1 °C and 12 h light/dark control with free access to food and water. Rat cages were cleaned every 7 days. All surgical procedures were performed under general anesthesia by intramuscular injection of ketamine (200 mg/kg, Alfasan Co., Woerden, Netherlands) and xylazine (10 mg/kg, Alfasan Co., Woerden, Netherland). The hair of the wound area of all animals was completely shaved, and then two wide circular wounds were created with a diameter of 2 cm. The distance between the two wounds was 4 cm.

### 4.13. Grouping and Treatment

Forty-five rats were randomly divided into five separate groups, with nine rats in each group. The five treatment groups were as follows:

Negative control group: wound was induced in animals, but no treatment was performed.

Standard treatment group: animals were treated with alpha ointment.

Positive control group: animals were treated with carboxymethyl cellulose powder gel (basal gel) without BSE.

7 d BSE gel group: animals were treated with CMC gel containing BSE from 7 days after arm amputation.

14 d BSE gel group: animals were treated with CMC gel containing BSE from 14 days after arm amputation.

An amount of 0.5 mL of gel and ointment was applied topically to the wounds daily. Furthermore, each group was divided into three subgroups. These three subgroups were ethically euthanized 7 d, 14 d and 21 d after the onset of treatment. The wound area was sampled in these three subgroups and used for further analysis.

### 4.14. Macroscopic Examination

Images were taken of the wounds on days 0, 3, 7, 10, 14, 18, and 21 using a smartphone camera (Samsung S8 plus, Seoul, Republic of Korea). A scaled ruler was placed next to each photo for calibration. The wound surface area was calculated by ImageJ software (Fiji-ImageJ x 64, V1.52p, US National Institutes of Health, Bethesda, MD, USA). The percentage of healing surface area was calculated using the following formula:

Wound healing ratio (%) = 100 × (specific day wound size/initial wound size)

### 4.15. Histological Examination

The tissue sample was removed from the wound area and placed in a sampling container containing 10% formalin buffer solution. After fixing the specimens, tissue sections were prepared by standard tissue preparation protocol and then stained with hematoxylin and eosin and Masson’s trichrome staining. The specimens were observed using a light microscope (Olympus, Tokyo, Japan)and photographed using a camera (Olympus, Tokyo, Japan) under 40×, 100×, 200× and 400× magnifications. In histopathological examination, the rate of acute and chronic inflammation, the amount of collagen, granulation tissue, fibroblast maturation, re-epithelization, and angiogenesis were scored qualitatively (Table 1). The scoring system was adopted with slight modification in scoring grade and protocol rom [103].

### 4.16. Measurement of Hydroxyproline

The amount of hydroxyproline in the wound area was measured using a hydroxyproline measuring kit (Kiazist Co., Hamedan, Iran). The protocol was performed according to the manufacturer’s instructions. Briefly, the tissue samples were first taken out of the freezer. Then, 20 to 40 mg of the sample was cut and placed in a microtube, and 100 μL of deionized water was poured into the microtubes before being homogenized by the homogenizer. Then, 100 μL of HCl (12 M) was added to the microtube and incubated at 120 °C for 3 to 4 h. The sample was then placed at 90 °C to evaporate the liquids. Then, 50 μL of assay buffer was poured into each microtube and then mixed. Then, 30 mg of activated charcoal was added to each sample and was thoroughly stirred. It was then centrifuged at 12,000× *g* for 15 min. The supernatant was used for further analysis. Each well of the kit was filled with 20 mL of the supernatant and detection solutions and then the absorption rate of the wells at a wavelength of 540–560 nm was read by spectrophotometry (Unicam ultraviolet visible spectrometry, Thermo Scientific, Waltham, MA, USA), and the concentration of the samples was obtained, and a comparison drawn with a standard curve.

### 4.17. Statistical Analysis

All statistical analyses were performed using IBM SPSS Statistics 26 (SPSS for Windows, version 26, SPSS Inc, Chicago, IL, USA) and GraphPad Prism (v7.0a, GraphPad Software, Inc., San Diego, CA, USA). Data were evaluated by one-way ANOVA and post hoc Tukey or LSD tests. The results are reported as mean ± standard error of the mean. The data were considered statistically significant at *p* ≤ 0.05.

## 5. Conclusions

The 7 and 14 d post arm wound induction hydro-alcoholic extracts of Persian Gulf brittle star, *O. cynthiae*, revealed significant wound healing potential in vitro on HFF cells and in vivo on a rat wound healing model. The Western blot analysis showed an increase in the expression of proliferative proteins, and computational analysis demonstrated a strong affinity between compounds of BSE and proteins. Moreover, the BSEs had higher effects on wound repair of rat wound models in histopathological scoring analysis. The results also demonstrated that collagen and hydroxyproline content increased during treatment with BSEs gels. However, during collagen excess, no scar was formed, due to the antifibrotic compounds present in the BSEs.

## Figures and Tables

**Table 1:** GC-MS compounds in ethanol extract from brittle star undergoing arm regeneration taken at different times after amputation.

Compounds	Formula	Effects	MW	Brittle Star Extracts						References
				0 h	3 h	3 d	5 d	7 d	14 d	
Cholesterol	C_27_H_46_O	Cell proliferative Contraction	386	+	-	-	-	+	-	[31]
Pseduosarsasapogenin-5,20-dien methyl ether	C_28_H_44_O_3_	Neuroregeneration	428	+	-	+	-	-	-	[32]
Ethyl.alpha-d-glucopyranoside	C_8_H_16_O_6_	Cell proliferative ECM production	208	-	-	-	-	+	-	[33]
Octadecanoic acid, methyl ester (Methyl stearate)	C_19_H_38_O_2_	Cell migration	298	-	-	-	-	-	+	[34]
Desulphosinigrin	C_10_H_17_NO_6_S	Antibacterial Anti-proliferation	279	+	-	-	-	-	-	[35,36]
5α-cholestan-3-one	C_29_H_50_O_2_	Contraction	430	+	-	+	-	-	-	[37]
Methoxyacetic acid,4-tetradecyl ester	C_17_H_34_O_3_	Antimicrobial	286	+	-	-	-	-	-	[38]
Hexadecanoic acid, ethyl ester	C_18_H_36_O_2_	Antimicrobial Anti-inflammatory	284	+	-	-	+	+	-	[39,40]
Pentadecanoic acid, ethyl ester	C_17_H_34_O_2_	Anti-inflammatory	270	+	-	-	-	-	-	[41]
1-Dodecanol,3,7,11-trimethyl-	C_15_H_32_O	Antimicrobial Anti-proliferation	228	+	-	+	-	-	-	[42,43]
Estra-1,3,5(10)-trien-17.beta.-ol	C_18_H_24_O	Antioxidant	256	+	-	-	-	-	-	[44]
9-Hexadecenoic acid, methyl ester(z)	C_17_H_32_O_2_	Antioxidant Antimicrobial	268	+	-	-	-	-	-	[45,46]
Heptadecanoic acid, ethyl ester	C_19_H_38_O_2_	Antioxidant	298	+	-	-	-	-	-	[47]
Dihydrovallesiachotamine	C_21_H_24_N_2_O_3_	Anti-proliferation Anti-inflammatory	352	+	-	+	-	-	-	[47,48,49]
Linoleic acid ethyl ester	C_20_H_36_O_2_	Antioxidant Anti-inflammatory	308	+	-	+	-	-	-	[50,51]
1-Monolinolenoyl-rac-glycerol	C_21_H_36_O_4_	Anti-inflammatory	352	+	-	+	-	-	-	[52]
Ethyl arachidonate	C_22_H_36_O_2_	Anti-proliferation	332	+	-	+	-	-	-	[53]
Tridocosahexaenoyl-glycerol	C_69_H_98_O_6_	Anti-inflammatory	1022	+	-	+	-	-	-	[54]
Ethyl iso-allocholate	C_26_H_44_O_5_	Anti-inflammatory Antimicrobial	436	+	-	+	-	-	-	[55]
1-Decanol,2,2-dimethyl-	C_12_H_26_O	Anti-proliferation	186	+	-	-	-	-	-	[56]
1-Hexadecanol,2-methyl-	C_17_H_36_O	Antimicrobial	256	+	-	-	-	-	-	[57]
1-Octanol, 2-butyl-	C_12_H_26_O	Antimicrobial	186	+	-	-	-	-	-	[58]
3-Hydroxyspirost-8-en-11-one	C_27_H_40_O_4_	Anti-inflammatory	428	+	-	+	+	-	-	[59]
Acridin-1(2H)-one,3,4-dihydro-3,3-dimethyl-9-propylamino-	C_18_H_22_N_2_O	Anti-inflammatory	282	-	+	-	-	-	-	[60]
Colchicine, N-desacetyl-N-[4-acetoxy-3,5-dimethoxycinnamoyl]	C_33_H_35_NO_10_	Antimicrobial Anti-proliferation	605	-	+	-	-	-	-	[61,62]
Pyridine-3-carboxylic acid,1,4-dihydro-5-cyano-2-hydroxy-4-(4-isopropylphenyl)-6-methyl-,ethyl ester	C_19_H_22_N_2_O_3_	Anti-inflammatory	326	-	+	-	-	-	-	[63]
Astaxanthin	C_40_H_52_O_4_	Anti-inflammatory Antioxidant	596	-	-	-	+	-	-	[64]
Octadecanoic acid, 2-hydroxy-1-(hydroxymethyl)ethyl ester	C_21_H_42_O_4_	Antimicrobial	358	-	-	-	+	+	+	[65]
Hexadecanoic acid, 2-hydroxy-1-(hydroxymethyl)ethyl ester	C_19_H_38_O_4_	Antimicrobial Antioxidant	330	-	-	-	+	+	-	[65,66]
n-Hexadecanoic acid	C_16_H_32_O_2_	Antimicrobial	256	-	-	-	+	+	-	[67]
7,9-Di-tert-butyl-1-oxaspiro(4,5)deca-6,9-diene-2,8-dione	C_17_H_24_O_3_	Antimicrobial	276	-	-	-	+	+	-	[68]
2-Heptanone, 6-(3-acetyl-1-cyclopropen-1-yl)-3-hydroxy-6-methyl-	C_13_H_20_O_3_	Antimicrobial	224	-	-	-	+	-	-	[69]
Tetradecanoic acid	C_14_H_28_O_2_	Antimicrobial	228	-	-	-	-	+	-	[70]
Oleic Acid	C_18_H_34_O_2_	Anti-inflammatory Antimicrobial	282	-	-	-	-	+	-	[71,72]
Dodecanal (Lauraldehyde)	C_12_H_24_O	Antimicrobial	184	-	-	-	-	-	+	[73,74]
1,2-Benzenedicarboxylic acid, bis(2-methylpropyl) ester	C_16_H_22_O_4_	Anti-proliferation Antifibrotic	278	-	-	-	-	-	+	[70]
7,9-Di-tert-butyl-1-oxaspiro(4,5)deca-6,9-diene-2,8-dione	C_17_H_24_O_3_	Antimicrobial	276	-	-	-	-	-	+	[68]
Hexadecanoic acid, methyl ester	C_17_H_34_O_2_	Antifibrotic Anti-inflammatory	270	-	-	-	-	-	+	[75]
Benzenepropanoic acid, 3,5-bis(1,1-dimethylethyl)-4-hydroxy-, methyl ester	C_18_H_28_O_3_	Antioxidant	292	-	-	-	-	-	+	[76]
1,3-Dioxolane, 2-(2,3-dimethyl-1-cyclopenten-3-yl)-2,4,5-trimethyl-	C_13_H_22_O_2_	Antimicrobial	210	-	-	-	-	-	+	[77]
Ethanol, 2,2′-(dodecylimino)bis, N-Dodecyldiethanolamine	C_16_H_35_NO_2_	Antimicrobial	273	-	-	-	-	-	+	[78]
2-(2-Azepan-1-yl-2-oxoethyl)-1-hydroxy-1-phenyl-octahydro-pyrido [1,2-a]azepin-4-one	C_24_H_34_N_2_O_3_	NA	398	+	-	+	-	-	-	-

NA, no data. +, shows presence and -, shows absence of the molecules in the extract.

**Table 2:** Criteria for the histological scoring of skin tissue sections were used for the evaluation of the healing effects of extract from brittle star undergoing arm regeneration on wound healing in rats.

Score	Parameter	Criteria
1	Collagen deposition	None
	Cell maturation	Unmatured cell
	Epithelialization	None to very minimal
	Cellular content	Mostly acute and chronic inflammatory cells
	Granulation tissue	None to sparse amount at wound edges
2	Collagen deposition	None
	Cell maturation	Mostly unmatured
	Epithelialization	Minimal to moderate
	Cellular content	Predominantly acute cells and few fibroblasts
	Granulation tissue	No layer to thin layer at wound center, thicker at wound edges
3	Collagen deposition	Few collagen fibers
	Cell maturation	Few maturated
	Epithelialization	Completely epithelialized, thin layer
	Cellular content	More fibroblast. Moderate inflammatory cells
	Granulation tissue	Thicker layer at wound center
4	Collagen deposition	More collagen fibers
	Cell maturation	Mild maturation
	Epithelialization	Thicker epithelial layer
	Cellular content	Predominantly fibroblasts
	Granulation tissue	Uniformly thick
	Collagen deposition	Moderate to extensive collagen deposited. Less mature than score 5
5	Cell maturation	Moderate maturation
	Epithelialization	Thick epithelium
	Cellular content	Fewer number of fibroblasts in dermis
	Granulation tissue	Uniformly thick
	Collagen deposition	Dense, organized, oriented collagen fibers
	Cell maturation	Maturation near normal skin tissue

**Table 3:** Criteria for the histological scoring of skin tissue sections were used for evaluation of the healing effects of extract from brittle star undergoing arm regeneration on wound healing in rats.

Ligand	α-SMA	E-cadherin	MMP9	MMP2	PTEN	AkT	pAkT	Cdk2	Cdk4	Cdk6	Cyclin D1	Cyclin E	pRb	p21	p27	p85α	p110	mTOR	p70S6K	4EBP1
1,2-Benzenedicarboxylic acid, bis(2-methylpropyl) ester	−5.1	−5	−6.9	−5.8	−5.6	−6.7	−6.5	−6.5	−6.6	−6.3	−6.2	−5.7	−5.8	−4.1	−5.2	−5.6	−6.3	−6.1	−6.4	−3.9
5α-cholestan-3-one	−6.6	−5.8	−7.5	−6.8	−7	−7.8	−8.1	−7.4	−6	−7.6	−7.3	−6.1	−6.3	−4.4	−6	−6.8	−7.7	−6.7	−7.3	−5
9-Hexadecenoic acid, methyl ester(z)	−4.1	−3.5	−6.3	−4.9	−5	−5.2	−5.2	−5.5	−5.8	−4.8	−4.6	−4.6	−5.1	−3	−4.8	−4.7	−4.9	−5	−5.3	−3.3
Astaxanthin	−7.9	−7.2	−9.1	−8.7	−9.3	−9	−10.6	−9.2	−8.2	−7.7	−9	−8.8	−8.8	−6	−8.3	−7.6	−8.8	−8.7	−8.5	−6.2
Benzenepropanoic acid, 3,5-bis(1,1-dimethylethyl)-4-hydroxy-, methyl ester	−5.3	−5.6	−6.5	−5.9	−6.2	−6.6	−7.3	−6.3	−6.8	−5.8	−6.2	−6	−6.3	−4	−5.3	−5.7	−7.2	−6.3	−6.8	−4.5
Cholesterol	−6.9	−6	−7.4	−6.6	−7.2	−7.4	−8.7	−8.3	−6	−7.8	−7	−6.8	−7.9	−4.6	−7.2	−6.9	−8.7	−6.2	−7.4	−5
Estra-1,3,5(10)-trien-17.beta.-ol	−6.7	−6.5	−7.4	−6.6	−7.1	−8.2	−9.4	−7.7	−8	−7.9	−8.3	−7.2	−7.4	−5.3	−6.5	−6.8	−8.7	−7.3	−8.5	−5.5
Ethyl.alpha-d-glucopyranoside	−5.3	−4.7	−5.6	−6	−5.1	−5.5	−5.5	−5.7	−5.3	−5.8	−5.5	−5.4	−5.5	−3.6	−4.1	−4.9	−5.5	−4.6	−5	−3.5
Heptadecanoic acid, ethyl ester	−4.1	−3.7	−6.4	−4.7	−4.8	−4.6	−5.2	−5.5	−5.7	−4	−4.5	−4.6	−4.6	−3.2	−5.1	−4.4	−4.9	−5.2	−4.3	−3
Hexadecanoic acid, methyl ester	−4.4	−4	−6.3	−5	−5.1	−4.8	−5.6	−5.4	−5.4	−4.8	−5.1	−5.1	−5.3	−2.9	−4.6	−4.3	−5.4	−5.1	−5	−3.4
Hexadecanoic acid, 2-hydroxy-1-(hydroxymethyl)ethyl ester	−4	−4	−6.2	−4.7	−4.8	−4.6	−5.1	−5.3	−5.3	−4.1	−4.8	−4.6	−4.3	−2.8	−4.6	−4.1	−4.4	−4.6	−4.7	−3.2
Linoleic acid ethyl ester	−4.9	−3.8	−5.4	−4.9	−5.2	−5.8	−5.8	−6	−4.5	−4.5	−5	−4.7	−4.9	−3.3	−4.9	−4.4	−5.3	−5.1	−4.8	−3.3
Octadecanoic acid, methyl ester (Methyl stearate)	−4.1	−3.6	−6.1	−5.3	−5	−5.1	−5.3	−5.7	−5	−4.1	−4.8	−4.4	−4.3	−3.5	−4.5	−4.5	−4.8	−5.2	−4.7	−2.9
Pseduosarsasapogenin-5,20-dien methyl ether	−7.3	−7.1	−7.4	−7.4	−7.5	−9	−9.6	−8.2	−7.8	−7.8	−7.2	−6.9	−7.5	−5.4	−6.9	−6.5	−8.6	−7.7	−7.8	−5.4

## Data Availability

Data are contained within the article or Supplementary Materials. Datasets related to this project can be obtained from the corresponding author upon reasonable request.

## Supplementary Materials

The following are available online at https://www.mdpi.com/article/10.3390/md21070381/s1, Figure S1: *Ophiocoma cynthiae* morphology, Figure S2: *Ophiocoma cynthiae* macroscopic evaluation. (A) Granules on dorsal surface of the brittle star; (B) Interradial margin; (C) Arm spines; (D) Oral frame, jaws, oral shields, adoral shields, oral papillae, and tooth papillae; (E) Oral surface of arm and tentacles; (F) Aboral surface of arm. All scales are 500 µm, Figure S3: GC-MS analysis of the 0 h and 3 h extracts of *Ophiocoma cynthiae* after arm amputation, Figure S4: GC-MS analysis of the 3rd day extract of *Ophiocoma cynthiae* after arm amputation, Figure S5: GC-MS analysis of the 5th day extracts of *Ophiocoma cynthiae* after arm amputation, Figure S6: GC-MS analysis of the 7th day extracts of *Ophiocoma cynthiae* after arm amputation, Figure S7: GC-MS analysis of the 14th day extracts of Ophiocoma cynthiae after arm amputation, Figure S8: The original gels of Western blot analysis.

## References

[1] Watt F.M. (2014). Mammalian skin cell biology: At the interface between laboratory and clinic. Science.

[2] Kanwar A. (2018). Skin barrier function. Indian J. Med. Res..

[3] Shedoeva A., Leavesley D., Upton Z., Fan C. (2019). Wound healing and the use of medicinal plants. Evid. Based Complement. Alternat. Med..

[4] Grubbs H., Manna B. (2018). Wound Physiology.

[5] Borena B.M., Martens A., Broeckx S.Y., Meyer E., Chiers K., Duchateau L., Spaas J.H. (2015). Regenerative skin wound healing in mammals: State-of-the-art on growth factor and stem cell based treatments. Cell. Physiol. Biochem..

[6] Rowan M.P., Cancio L.C., Elster E.A., Burmeister D.M., Rose L.F., Natesan S., Chan R.K., Christy R.J., Chung K.K. (2015). Burn wound healing and treatment: Review and advancements. Crit. Care.

[7] Morgan E.D., Bledsoe S.C., Barker J. (2000). Ambulatory management of burns. Am. Fam. Physician.

[8] Singh S., Young A., McNaught C.-E. (2017). The physiology of wound healing. Surgery.

[9] Zhang X., Shu W., Yu Q., Qu W., Wang Y., Li R. (2020). Functional biomaterials for treatment of chronic wound. Front. Bioeng. Biotechnol..

[10] Heng M.C. (2011). Wound healing in adult skin: Aiming for perfect regeneration. Int. J. Dermatol..

[11] Schiavon M., Francescon M., Drigo D., Salloum G., Baraziol R., Tesei J., Fraccalanza E., Barbone F. (2016). The use of Integra dermal regeneration template versus flaps for reconstruction of full-thickness scalp defects involving the calvaria: A cost–benefit analysis. Aesthetic Plast. Surg..

[12] Boyce S.T., Lalley A.L. (2018). Tissue engineering of skin and regenerative medicine for wound care. Burn. Trauma.

[13] Pereira R.F., Bartolo P.J. (2016). Traditional therapies for skin wound healing. Adv. Wound Care.

[14] Poss K.D. (2010). Advances in understanding tissue regenerative capacity and mechanisms in animals. Nat. Rev. Genet..

[15] Carnevali M.C. (2006). Regeneration in Echinoderms: Repair, regrowth, cloning. Invertebr. Surviv. J..

[16] Czarkwiani A., Ferrario C., Dylus D.V., Sugni M., Oliveri P. (2016). Skeletal regeneration in the brittle star *Amphiura filiformis*. Front. Zool..

[17] Biressi A.C.M., Zou T., Dupont S., Dahlberg C., Di Benedetto C., Bonasoro F., Thorndyke M., Carnevali M.D.C. (2010). Wound healing and arm regeneration in *Ophioderma longicaudum* and *Amphiura filiformis* (Ophiuroidea, Echinodermata): Comparative morphogenesis and histogenesis. Zoomorphology.

[18] Ben Khadra Y., Ferrario C., Di Benedetto C., Said K., Bonasoro F., Candia Carnevali M.D., Sugni M. (2015). Wound repair during arm regeneration in the red starfish *E chinaster sepositus*. Wound Repair Regen..

[19] Dai Y., Prithiviraj N., Gan J., Zhang X.A., Yan J. (2016). Tissue extract fractions from starfish undergoing regeneration promote wound healing and lower jaw blastema regeneration of zebrafish. Sci. Rep..

[20] Abolhasani I., Baharara J., Mahdavi S.N., Amini E. (2020). The regenerative properties of the extracted polysaccharide from Brittle star (*Ophiocoma erinaceus*) on cutaneous wound in male Wistar rat. Nova Biol. Reper..

[21] Afzali M., Baharara J., Shahrokhabadi K., Amini E. (2015). Effect of the Persian Gulf Brittle Star (*Ophiocoma erinaceus*) dichloromethane extract on induction of apoptosis on EL4 Cell Line. J. Rafsanjan Univ. Med. Sci..

[22] Amini E., Nabiuni M., Baharara J., Parivar K., Asili J. (2015). Metastatic inhibitory and radical scavenging efficacies of saponins extracted from the brittle star (*Ophiocoma erinaceus*). Asian Pac. J. Cancer Prev..

[23] Amini E., Nabiuni M., Baharara J., Parivar K., Asili J. (2017). In-vitro pro apoptotic effect of crude saponin from *Ophiocoma erinaceus* against cervical cancer. Iran. J. Pharm. Res..

[24] Baharara J., Amini E. (2015). Phytochemical screening, antioxidant effect and down regulation of TGF-β induced by *Ophiocoma erinaceus* Brittle star crude extract. Zahedan J. Res. Med. Sci..

[25] Baharara J., Amini E., Namvar F. (2016). Evaluation of the anti-proliferative effects of *Ophiocoma erinaceus* methanol extract against human cervical cancer cells. Avicenna J. Med. Biotechnol..

[26] Baharara J., Amini E., Salek-Abdollahi F. (2020). Anti-inflammatory properties of saponin fraction from *Ophiocoma erinaceus*. Iran. J. Fish. Sci..

[27] Baharara J., Mahdavi–Shahri N., Shaddel N. (2014). The local effect of Persian Gulf brittle star (*Ophiocoma erinaceus*) alcoholic extract on cutaneous wound healing in Balb/C mouse. J. Birjand Univ. Med. Sci..

[28] Benavides-Serrato M., O’Hara T.D. (2008). A new species in the *Ophiocoma erinaceus* complex from the South-west Pacific Ocean (Echinodermata: Ophiuroidea: Ophiocomidae). Mem. Mus. Vic..

[29] Olbers J.M. (2016). Taxonomy, biodiversity and biogeography of the brittle stars (Echinodermata: Ophiuroidea) of South Africa; Thesis manuscript. OpenUSCT.

[30] Pomory C.M. (2007). Key to the common shallow-water brittle stars (Echinodermata: Ophiuroidea) of the Gulf of Mexico and Caribbean Sea. Caribb. J. Sci..

[31] Tyagi S.C., Kumar S., Katwa L. (1997). Differential regulation of extracellular matrix metalloproteinase and tissue inhibitor by heparin and cholesterol in fibroblast cells. J. Mol. Cell. Cardiol..

[32] Barraclough P., Hanson J., Gunning P., Rees D., Xia Z., Hu Y. 5-Beta-Sapogenin and Pseudosapogenin Derivatives and Their Use in the Treatment of Dementia.

[33] Bogaki T., Mitani K., Oura Y., Ozeki K. (2017). Effects of ethyl-α-d-glucoside on human dermal fibroblasts. Biosci. Biotechnol. Biochem..

[34] Liu Y., Xu L., Hu L., Chen D., Yu L., Li X., Chen H., Zhu J., Chen C., Luo Y. (2020). Stearic acid methyl ester promotes migration of mesenchymal stem cells and accelerates cartilage defect repair. J. Orthop. Transl..

[35] Sosa A.A., Bagi S.H., Hameed I.H. (2016). Analysis of bioactive chemical compounds of *Euphorbia lathyrus* using gas chromatography-mass spectrometry and fourier-transform infrared spectroscopy. J. Pharmacogn. Phytother..

[36] Krishnaveni M. (2015). Docking, Simulation Studies of Desulphosinigrin–Cyclin Dependent Kinase 2, an Anticancer Drug Target. Int. J. Pharm. Sci. Rev. Res..

[37] Sytchev V.I., Odnoshivkina Y.G., Ursan R.V., Petrov A.M. (2017). Oxysterol, 5α-cholestan-3-one, modulates a contractile response to β2-adrenoceptor stimulation in the mouse atria: Involvement of NO signaling. Life Sci..

[38] Agnel R., Mohan V. (2014). GC–MS analyses of bioactive compounds present in the whole plant of *Andrographis echioides* (L.) nees. Eur. J. Biomed. Pharm. Sci..

[39] Policegoudra R., Chattopadhyay P., Aradhya S., Shivaswamy R., Singh L., Veer V. (2014). Inhibitory effect of *Tridax procumbens* against human skin pathogens. J. Herb. Med..

[40] Saeed N.M., El-Demerdash E., Abdel-Rahman H.M., Algandaby M.M., Al-Abbasi F.A., Abdel-Naim A.B. (2012). Anti-inflammatory activity of methyl palmitate and ethyl palmitate in different experimental rat models. Toxicol. Appl. Pharmacol..

[41] Shareef I.M., Leelavathi S., Gopinath S. (2014). Isolation and Characterization of Pentadecanoic Acid Ethyl Ester from the Methanolic Extract of the Aerial Parts of *Anisomeles Malabarica* (L). R. BR. Glob. J. Res. Med. Plants Indig. Med..

[42] Esghaei M., Ghaffari H., Esboei B.R., Tapeh Z.E., Salim F.B., Motevalian M. (2018). Evaluation of anticancer activity of *Camellia sinensis* in the Caco-2 colorectal cancer cell line. Asian Pac. J. Cancer Prev..

[43] Togashi N., Shiraishi A., Nishizaka M., Matsuoka K., Endo K., Hamashima H., Inoue Y. (2007). Antibacterial activity of long-chain fatty alcohols against *Staphylococcus aureus*. Molecules.

[44] Osman N., Harun A. (2019). Antioxidative constituents from petroleum ether extract of *Curcuma longa* leaves. Gading J. Sci. Technol..

[45] Meechaona R., Sengpracha W., Banditpuritat J., Kawaree R., Phutdhawong W. (2007). Fatty acid content and antioxidant activity of Thai bananas. Maejo Int. J. Sci. Technol..

[46] Wei L.S., Wee W., Siong J.Y.F., Syamsumir D.F. (2011). Characterization of anticancer, antimicrobial, antioxidant properties and chemical compositions of *Peperomia pellucida* leaf extract. Acta Med. Iran..

[47] Vijayakumar K., Prasanna B., Rengarajan R., Rathinam A., Velayuthaprabhu S., Vijaya Anand A. (2020). Anti-diabetic and hypolipidemic effects of *Cinnamon cassia* bark extracts: An in vitro, in vivo, and in silico approach. Arch. Physiol. Biochem..

[48] Nisar A., Mamat A.S., Hatim M.I., Aslam M.S., Syarhabil M. (2016). An updated review on *Catharanthus roseus*: Phytochemical and pharmacological analysis. Ind. Res. J. Pharm. Sci..

[49] Cao P., Liang Y., Gao X., Li X.-M., Song Z.-Q., Liang G. (2012). Monoterpenoid indole alkaloids from *Alstonia yunnanensis* and their cytotoxic and anti-inflammatory activities. Molecules.

[50] Bae M.-S., Shin J.-S., Lee K.-Y., Lee K.-H., Kim Y.J. (2014). Long-range transport of biomass burning emissions based on organic molecular markers and carbonaceous thermal distribution. Sci. Total Environ..

[51] Kim C.-H., Lee M.-A., Kim T.-W., Jang J.Y., Kim H.J. (2012). Anti-inflammatory effect of *Allium hookeri* root methanol extract in LPS-induced RAW264. 7 cells. J. Korean Soc. Food Sci. Nutr..

[52] Idan S.A., Al-Marzoqi A.H., Hameed I.H. (2015). Spectral analysis and anti-bacterial activity of methanolic fruit extract of *Citrullus colocynthis* using gas chromatography-mass spectrometry. Afr. J. Biotechnol..

[53] Prabakaran R., Kumar T.S., Rao M. (2014). GC-MS Analysis and in vitro Cytotoxicity Studies of Root Bark Exudates of *Hardwickia binata* Roxb. Methods.

[54] Aswathy T., Gayathri E., Praveen J., Achuthsankar S.N., Sugunan V. (2019). Phytoprofiling of medicinal plant *Cayratia pedata* by qualitative and quantitative method. J. Pharmacogn. Phytochem..

[55] Hussein A.O., Mohammed G.J., Hadi M.Y., Hameed I.H. (2016). Phytochemical screening of methanolic dried galls extract of *Quercus infectoria* using gas chromatography-mass spectrometry (GC-MS) and Fourier transform-infrared (FT-IR). J. Pharmacogn. Phytother..

[56] Kreja L., Seidel H.-J. (2002). On the cytotoxicity of some microbial volatile organic compounds as studied in the human lung cell line A549. Chemosphere.

[57] Altaee N., Kadhim M.J., Hameed I.H. (2017). Characterization of metabolites produced by *E. coli* and analysis of its chemical compounds using GC-MS. Int. J. Curr. Pharm. Rev. Res..

[58] Setyati W.A., Pramesti R., Susanto A., Chrisna A., Zainuddin M. In vitro antibacterial study and spectral analysis of brown seaweed *Sargassum crassifolium* extract from Karimunjawa Islands, Jepara. Proceedings of the IOP Conference Series: Earth and Environmental Science.

[59] Altameme H.J., Hameed I.H., Abu-Serag N.A. (2015). Analysis of bioactive phytochemical compounds of two medicinal plants, *Equisetum arvense* and *Alchemila valgaris* seed using gas chromatographymass spectrometry and fourier-transform infrared spectroscopy. Malays. Appl. Biol..

[60] Lombardino J.G., Mylari B.L., Mcmanus J.M. (1996). Process for the Preparation of Therapeutically Useful Pyrroloquinoline, Benzothiazine, Acridine, Phenoxazine and Phenothiazine Derivatives.

[61] Gelmi M.L., Mottadelli S., Pocar D., Riva A., Bombardelli E., De Vincenzo R., Scambia G. (1999). N-deacetyl-N-aminoacylthiocolchicine derivatives: Synthesis and biological evaluation on MDR-positive and MDR-negative human cancer cell lines. J. Med. Chem..

[62] Kurek J., Kwaśniewska-Sip P., Myszkowski K., Cofta G., Murias M., Barczyński P., Jasiewicz B., Kurczab R. (2018). 7-Deacetyl-10-alkylthiocolchicine derivatives—New compounds with potent anticancer and fungicidal activity. MedChemComm.

[63] Beyett T.S., Gan X., Reilly S.M., Gomez A.V., Chang L., Tesmer J.J.G., Saltiel A.R., Showalter H.D. (2018). Design, synthesis, and biological activity of substituted 2-amino-5-oxo-5H-chromeno[2,3-b]pyridine-3-carboxylic acid derivatives as inhibitors of the inflammatory kinases TBK1 and IKKε for the treatment of obesity. Bioorganic Med. Chem..

[64] Kishimoto Y., Yoshida H., Kondo K. (2016). Potential anti-atherosclerotic properties of astaxanthin. Mar. Drugs.

[65] Nath K., Talukdar A.D., Bhattacharya M.K., Bhowmik D., Chetri S., Choudhury D., Mitra A., Choudhury N.A. (2019). *Cyathea gigantea* (Cyatheaceae) as an antimicrobial agent against multidrug resistant organisms. BMC Complement. Altern. Med..

[66] Tyagi T., Agarwal M. (2017). Phytochemical screening and GC-MS analysis of bioactive constituents in the ethanolic extract of *Pistia stratiotes L.* and *Eichhornia crassipes* (Mart.) solms. J. Pharmacogn. Phytochem..

[67] Aparna V., Dileep K.V., Mandal P.K., Karthe P., Sadasivan C., Haridas M. (2012). Anti-inflammatory property of n-hexadecanoic acid: Structural evidence and kinetic assessment. Chem. Biol. Drug Des..

[68] Monisha S.I., Vimala J.R. (2018). Extraction, Identification and Pharmacological Evaluation of Phyto-Active Compound in *Manilkara Hexandra* (Roxb.) Dubard Stem Bark. Biosci. Biotechnol. Res. Asia.

[69] Zhang D., Yu S., Yang Y., Zhang J., Zhao D., Pan Y., Fan S., Yang Z., Zhu J. (2020). Antifungal effects of volatiles produced by *Bacillus subtilis* against *Alternaria solani* in potato. Front. Microbiol..

[70] Liu C.-H., Huang H.-Y. (2012). Antimicrobial activity of curcumin-loaded myristic acid microemulsions against *Staphylococcus epidermidis*. Chem. Pharm. Bull..

[71] Carrillo Pérez C., Cavia Camarero M.d.M., Alonso de la Torre S. (2012). Role of oleic acid in immune system; mechanism of action; a review. Nutr. Hosp..

[72] Mudgil P., Whitehall J. (2014). Oleic acid as an Antibacterial for Treating Eye Infections. Investig. Ophthalmol. Vis. Sci..

[73] Hayashi K., Kamiya M., Hayashi T. (1995). Virucidal effects of the steam distillate from *Houttuynia cordata* and its components on HSV-1, influenza virus, and HIV. Planta Med..

[74] Huang J., Su D., Feng Y., Liu K., Song Y. (2014). Antiviral herbs-present and future. Infect. Disord.-Drug Targets.

[75] El-Demerdash E. (2011). Anti-inflammatory and antifibrotic effects of methyl palmitate. Toxicol. Appl. Pharmacol..

[76] Molander P., Haugland K., Hegna D., Ommundsen E., Lundanes E., Greibrokk T. (1999). Determination of low levels of an antioxidant in polyolefins by large-volume injection temperature-programmed packed capillary liquid chromatography. J. Chromatogr. A.

[77] Ovsyannikova M., Vol’eva V., Belostotskaya I., Komissarova N., Malkova A., Kurkovskaya L. (2013). Antibacterial activity of substituted 1, 3-Dioxolanes. Pharm. Chem. J..

[78] Chaouat C., Balor S., Roques C., Franceschi-Messant S., Perez E., Rico-Lattes I. (2013). Antimicrobial catanionic vesicular self-assembly with improved spectrum of action. J. Surfactants Deterg..

[79] Prabhu K., Bragadeeswaran S. (2013). Biological properties of brittle star *Ophiocnemis marmorata* collected from Parangipettai, Southeast coast of India. J. Microbiol. Antimicrob..

[80] Heber-Katz E. (2017). Oxygen, metabolism, and regeneration: Lessons from mice. Trends Mol. Med..

[81] Cao X., Wang Y., Wu C., Li X., Fu Z., Yang M., Bian W., Wang S., Song Y., Tang J. (2018). Cathelicidin-OA1, a novel antioxidant peptide identified from an amphibian, accelerates skin wound healing. Sci. Rep..

[82] Li B., Li J., Xu W.W., Guan X.Y., Qin Y.R., Zhang L.Y., Law S., Tsao S.W., Cheung A.L. (2014). Suppression of esophageal tumor growth and chemoresistance by directly targeting the PI3K/AKT pathway. Oncotarget.

[83] Hennessy B.T., Smith D.L., Ram P.T., Lu Y., Mills G.B. (2005). Exploiting the PI3K/AKT pathway for cancer drug discovery. Nat. Rev. Drug Discov..

[84] Lee D.-Y., Li Y.-S.J., Chang S.-F., Zhou J., Ho H.-M., Chiu J.-J., Chien S. (2010). Oscillatory flow-induced proliferation of osteoblast-like cells is mediated by αvβ3 and β1 integrins through synergistic interactions of focal adhesion kinase and Shc with phosphatidylinositol 3-kinase and the Akt/mTOR/p70S6K pathway. J. Biol. Chem..

[85] Yao X., Jiang W., Yu D., Yan Z. (2019). Luteolin inhibits proliferation and induces apoptosis of human melanoma cells in vivo and in vitro by suppressing MMP-2 and MMP-9 through the PI3K/AKT pathway. Food Funct..

[86] Ma J., Wang L., Li J., Zhang G., Tao H., Li X., Sun D., Hu Y. (2018). Swainsonine inhibits invasion and the EMT process in esophageal carcinoma cells by targeting twist1. Oncol. Res..

[87] Trimis G., Chatzistamou I., Politi K., Kiaris H., Papavassiliou A.G. (2008). Expression of p21waf1/Cip1 in stromal fibroblasts of primary breast tumors. Hum. Mol. Genet..

[88] Al-Ansari M., Hendrayani S., Shehata A., Aboussekhra A. (2013). p16 INK4A Represses the paracrine tumor-promoting effects of breast stromal fibroblasts. Oncogene.

[89] Lloyd R.V., Erickson L.A., Jin L., Kulig E., Qian X., Cheville J.C., Scheithauer B.W. (1999). p27kip1: A multifunctional cyclin-dependent kinase inhibitor with prognostic significance in human cancers. Am. J. Pathol..

[90] Tsoli E., Gorgoulis V.G., Zacharatos P., Kotsinas A., Mariatos G., Kastrinakis N.G., Kokotas S., Kanavaros P., Asimacopoulos P., Bramis J. (2001). Low levels of p27 in association with deregulated p53-pRb protein status enhance tumor proliferation and chromosomal instability in non-small cell lung carcinomas. Mol. Med..

[91] Kaproth-Joslin K.A., Li X., Reks S.E., Kelley G.G. (2008). Phospholipase Cδ1 regulates cell proliferation and cell-cycle progression from G1-to S-phase by control of cyclin E–CDK2 activity. Biochem. J..

[92] Xing Y., Ren S., Ai L., Sun W., Zhao Z., Jiang F., Zhu Y., Piao D. (2019). ZNF692 promotes colon adenocarcinoma cell growth and metastasis by activating the PI3K/AKT pathway. Int. J. Oncol..

[93] Graña X., Reddy E.P. (1995). Cell cycle control in mammalian cells: Role of cyclins, cyclin dependent kinases (CDKs), growth suppressor genes and cyclin-dependent kinase inhibitors (CKIs). Oncogene.

[94] Paydar S., Akrami M., Dehghanian A., Moghadam R.A., Heidarpour M., Khoob A.B., Dalfardi B. (2016). A Comparison of the Effects of Alpha and Medical-Grade Honey Ointments on Cutaneous Wound Healing in Rats. J. Pharm..

[95] Cissell D.D., Link J.M., Hu J.C., Athanasiou K.A. (2017). A modified hydroxyproline assay based on hydrochloric acid in Ehrlich’s solution accurately measures tissue collagen content. Tissue Eng. Part C Methods.

[96] Fatemi S., Jamili S., Valinassab T., Kuranlu N. (2010). Diversity of Ophiuroidea from lengeh portand Qeshm island in the Persian Gulf. J. Fish Aquat. Sci..

[97] Price A. (1983). Echinoderms of Saudi Arabia: Echinoderms of the Arabian Gulf Coast of Saudi Arabia. Fauna of Saudi Arabia.

[98] Heinzeller T., Nebelsick J.H. Echinoderms: Munchen. Proceedings of the 11th International Echinoderm Conference.

[99] Clark A.M. (1971). Monograph of shallow-water Indo-West Pacific echinoderms. Trust. Br. Mus. Nat. Hist. Publ..

[100] Stöhr S. (2011). New records and new species of Ophiuroidea (Echinodermata) from Lifou, Loyalty Islands, New Caledonia. Zootaxa.

[101] Giorgio G.D., Rubilar T., Brogger M.I. (2015). Histological analysis after arm tip amputation in the brittle star *Ophioplocus januarii* (Echinodermata: Ophiuroidea). Rev. De Biol. Trop..

[102] Zhang J.-Y., Tao L.-Y., Liang Y.-J., Chen L.-M., Mi Y.-J., Zheng L.-S., Wang F., She Z.-G., Lin Y.-C., To K.K.W. (2010). Anthracenedione derivatives as anticancer agents isolated from secondary metabolites of the mangrove endophytic fungi. Mar. Drugs.

[103] Sultana J., Molla M.R., Kamal M., Shahidullah M., Begum F., Bashar M.A. (2009). Histological differences in wound healing in maxillofacial region in patients with or without risk factors. Bangladesh J. Pathol..

