# Supplementary material for: Tissue Extract from Brittle Star Undergoing Arm Regeneration Promotes Wound Healing in Rat
Source: Mar Drugs. 2023 Jun 28;21(7):381. doi: 10.3390/md21070381 (PMC10381614; doi:10.3390/md21070381)
Supplement: Supplementary file 1 [file marinedrugs-21-00381-s001.zip › marinedrugs-2109249-supplementary/marinedrugs-2109249-supplementary.pdf]

Tissue Extract from Brittle Star Undergoing Arm Regeneration Promotes Wound Healing in Rat

Alireza Afshar [1,2,3,†], Arezoo Khoradmehr [3,†], Fariborz Nowzari [4,†], Neda Baghban [3,†], Masoud Zare [3,†], Maryam Najafi [4], Seyedeh Zahra Keshavarzi [4], Fatemeh Zendehboudi [2,3], Gholamhossein Mohebbi [3], Alireza Barmak [5], Fatemeh Mohajer [2,3], Nahid Basouli [2,3], Mohammadreza Keshtkar [2,3], Aida Iraji [6,7], Fatemeh Sari Aslani [8], Cambyz Irajie [9], Iraj Nabipour [3], Mehdi Mahmudpour [10], Nader Tanideh [4,11,*] and Amin Tamadon [1,12,*]

1 PerciaVista R&D Co., Shiraz 73, Iran
2 Student Research Committee, Bushehr University of Medical Sciences, Bushehr 75, Iran
3 The Persian Gulf Marine Biotechnology Research Center, The Persian Gulf Biomedical Sciences Research Institute, Bushehr University of Medical Sciences, Bushehr 75, Iran
4 Stem Cells Technology Research Center, Shiraz University of Medical Sciences, Shiraz 73, Iran
5 Food Lab, Bushehr University of Medical Sciences, Bushehr 75, Iran
6 Medicinal and Natural Products Chemistry Research Center, Shiraz University of Medical Sciences, Shiraz 73, Iran
7 Central Research Laboratory, Shiraz University of Medical Sciences, Shiraz 73, Iran
8 Molecular Dermatology Research Center, School of Medicine, Shiraz University of Medical Sciences, Shiraz 73, Iran
9 Department of Medical Biotechnology, School of Advanced Medical Sciences and Technologies, Shiraz University of Medical Sciences, Shiraz 73, Iran
10 The Persian Gulf Tropical Medicine Research Center, The Persian Gulf Biomedical Sciences Research Institute, Bushehr University of Medical Sciences, Bushehr 75, Iran
11 Department of Pharmacology, Medical School, Shiraz University of Medical Sciences, Shiraz 73, Iran
12 Department for Scientific Work, West Kazakhstan Marat Ospanov Medical University, Aktobe, 030012, Kazakhstan
* Correspondence: tanidehn@gmail.com (N.T.); amintamaddon@yahoo.com (A.T.); Tel.: +98-71-3234-1025 (N.T.); +7-705-629-9350 (A.T.)
† These authors contribute equally to this manuscript.

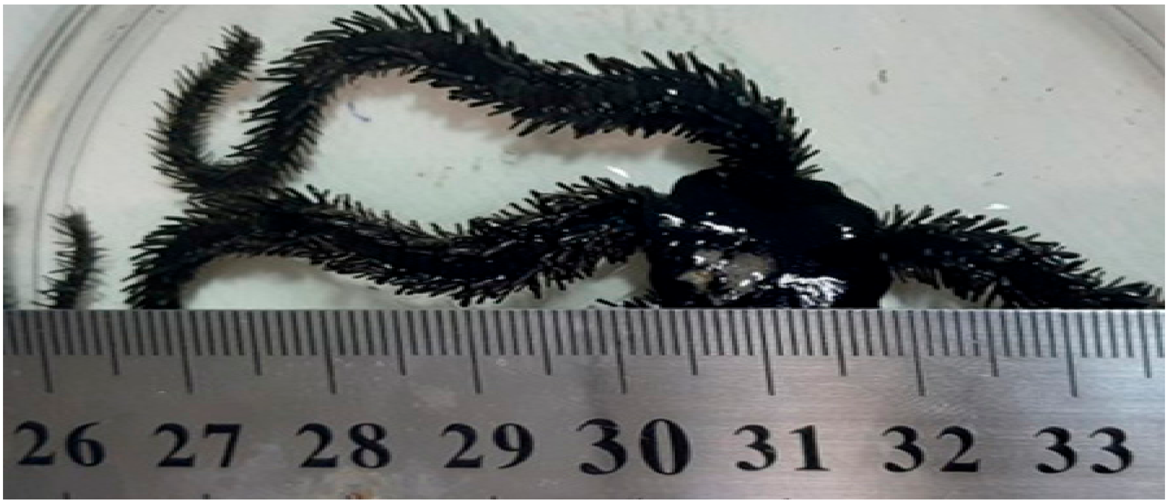

Figure S1. *Ophiocoma cynthiae* morphology.

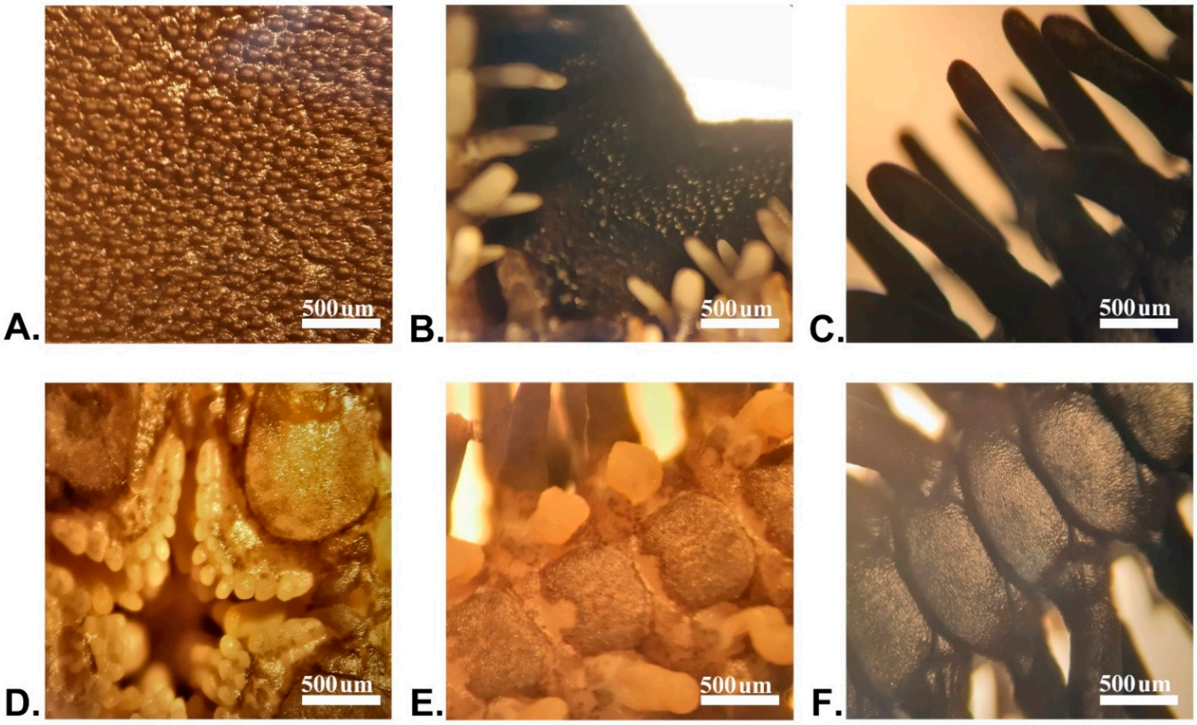

Figure S2. *Ophiocoma cynthiae* macroscopic evaluation. A. Granules on dorsal surface of the brittle star; B. Interradial margin; C. Arm spines; D. (D) Oral frame, jaws, oral shields, adoral shields, oral papillae, and tooth papillae; E. Oral surface of arm and tentacles; F. Aboral surface of arm. All scales are 500µm.

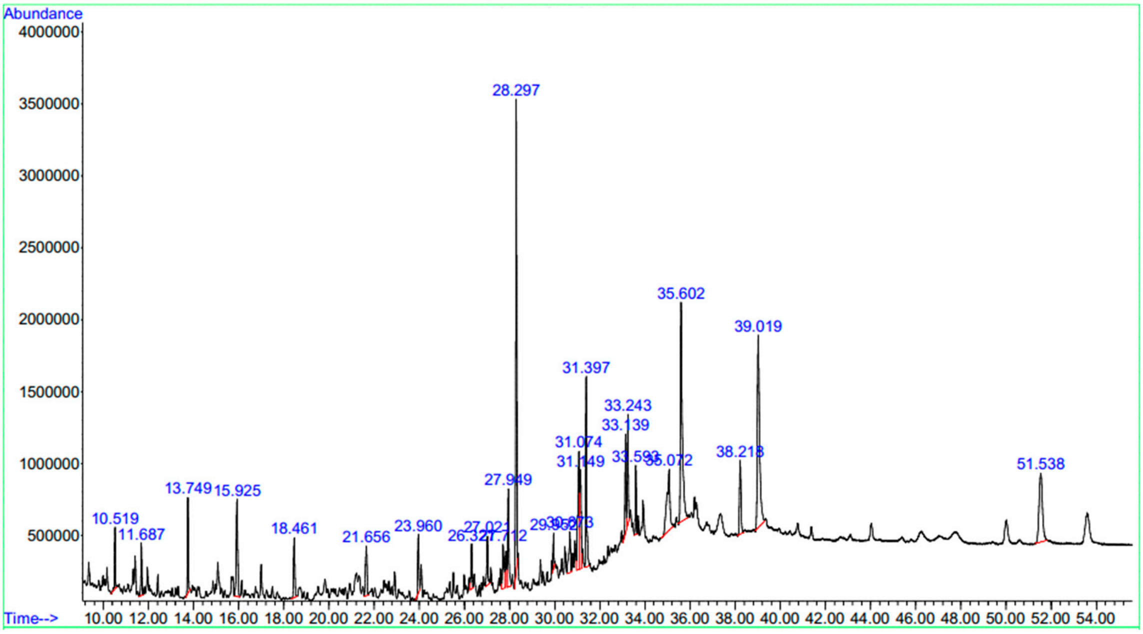

Figure S3. GC-MS analysis of the 0h and 3h extracts of *Ophiocoma cynthiae* after arm amputation.

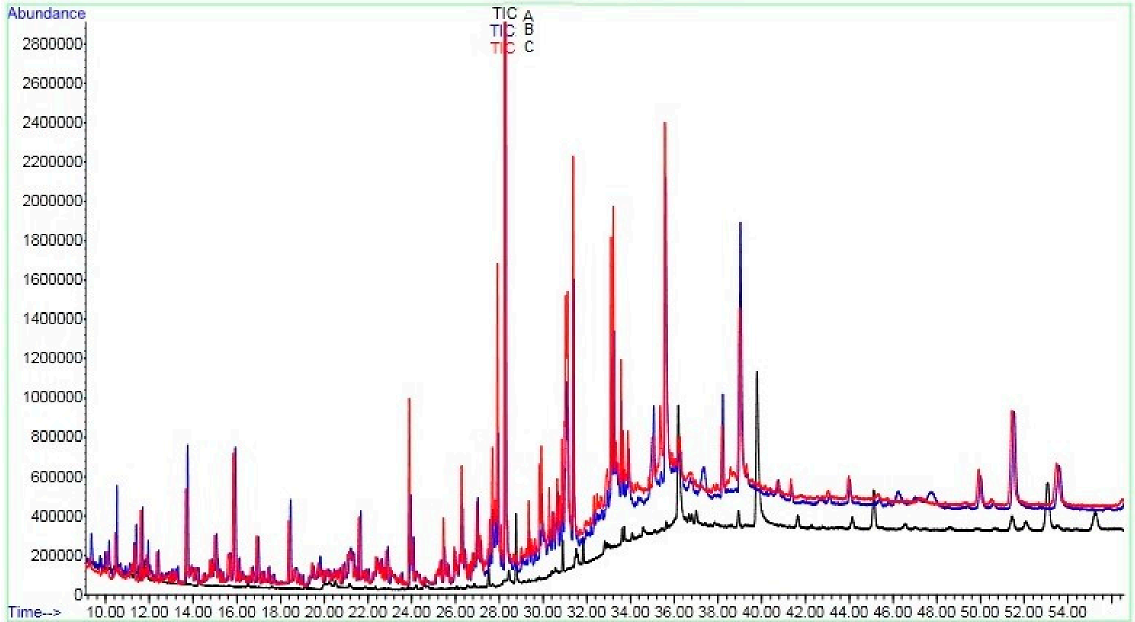

Figure S4. GC-MS analysis of the 3rd day extract of *Ophiocoma cynthiae* after arm amputation.

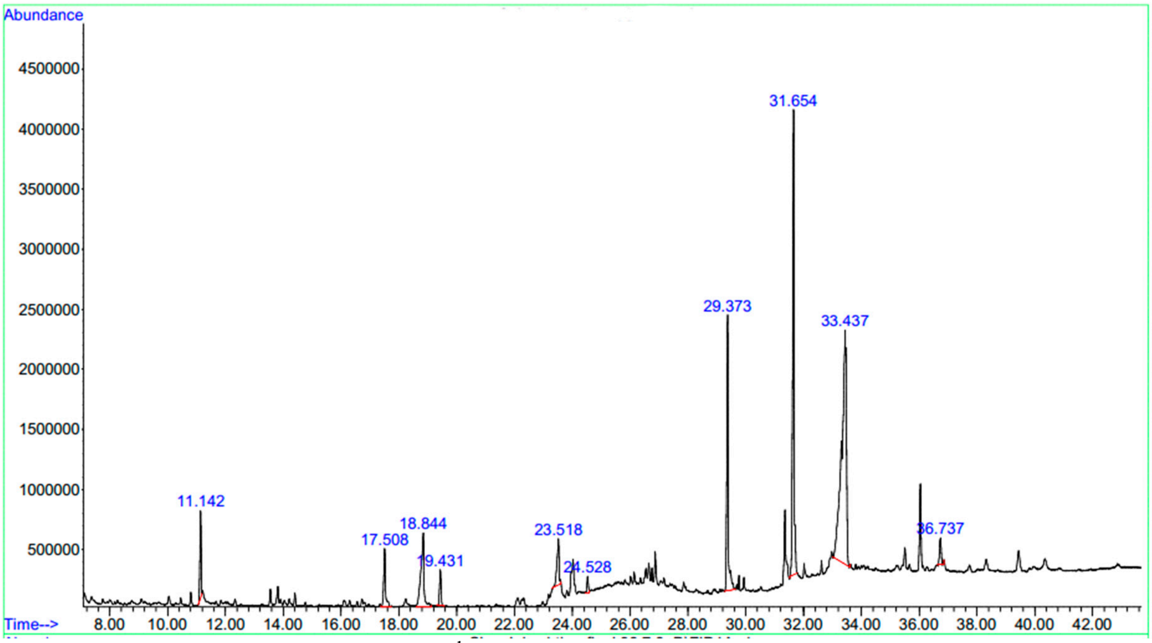

Figure S5. GC-MS analysis of the 5th day extracts of *Ophiocoma cynthiae* after arm amputation.

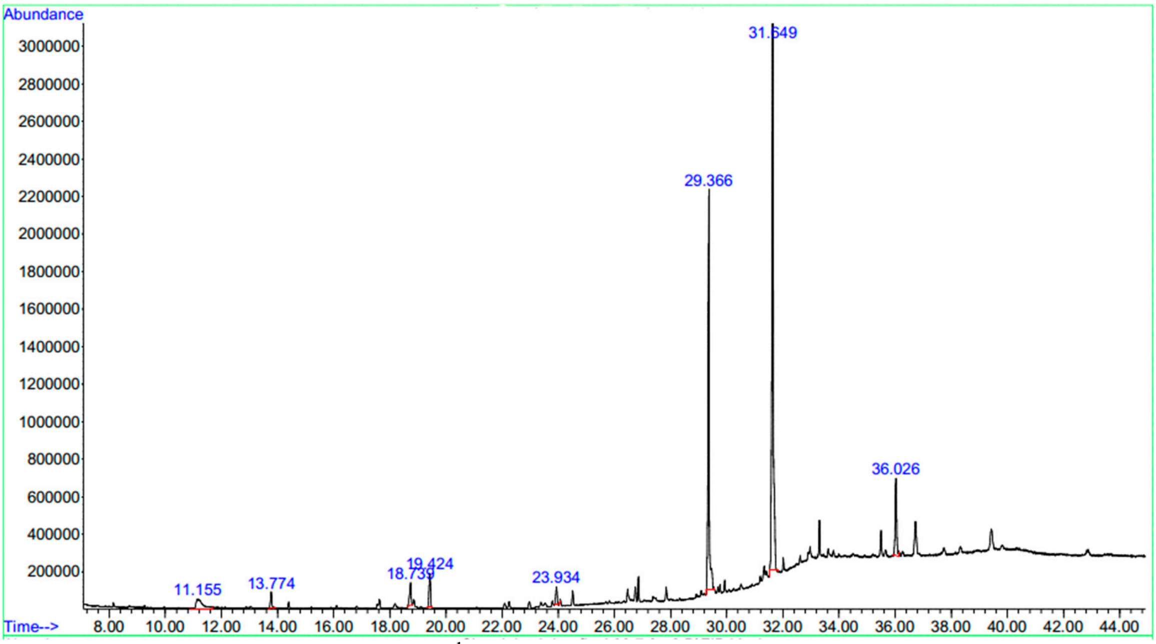

Figure S6. GC-MS analysis of the 7th day extracts of *Ophiocoma cynthiae* after arm amputation.

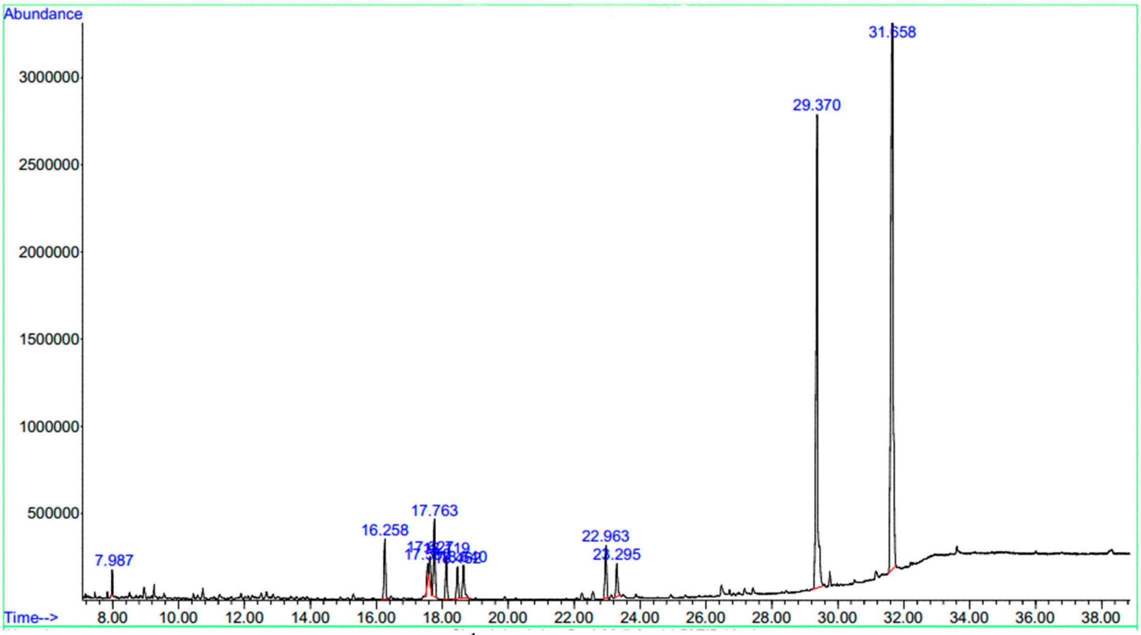

Figure S7. GC-MS analysis of the 14th day extracts of *Ophiocoma cynthiae* after arm amputation.

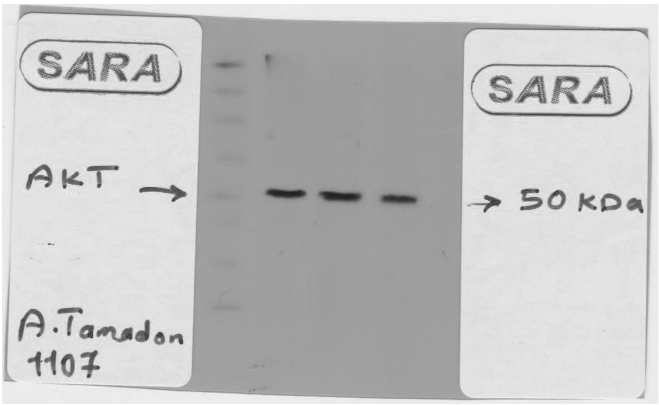
SARA
SARA
AKT →
→ 50 KDa
A.Tamadon
1107

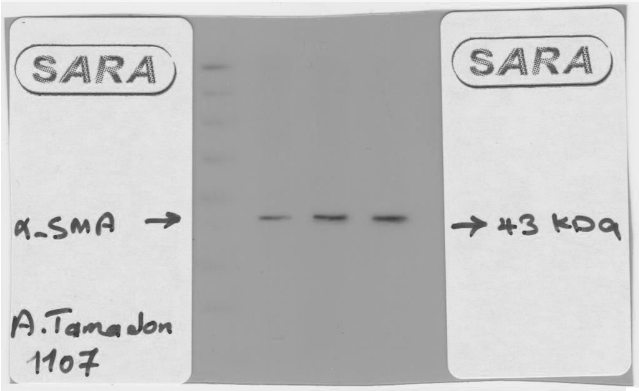
SARA
SARA
α-SMA →
→ 43 KDa
A.Tamadon
1107

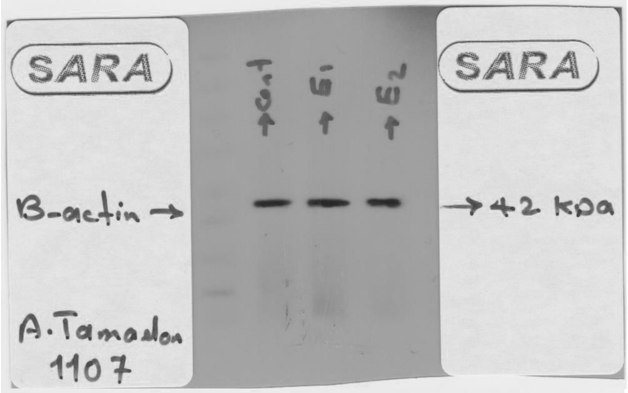
SARA
SARA
B-actin →
→ 42 KDa
A.Tamadon
1107

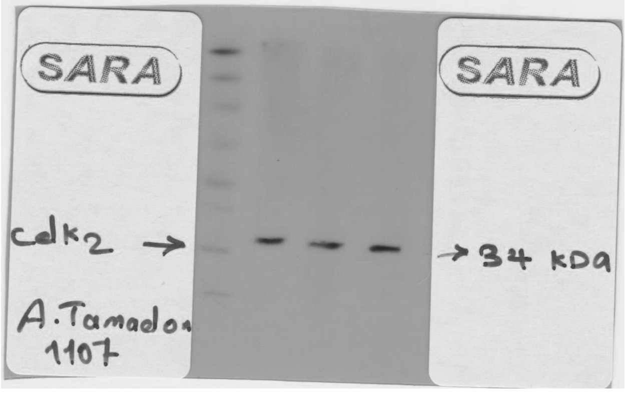
SARA
SARA
cdk2 →
→ 34 KDa
A.Tamadon
1107

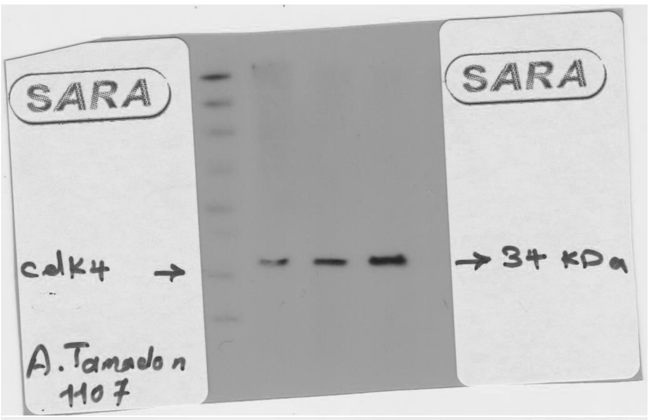
SARA
SARA
cdk4 →
→ 34 KDa
A. Tamadon
1107

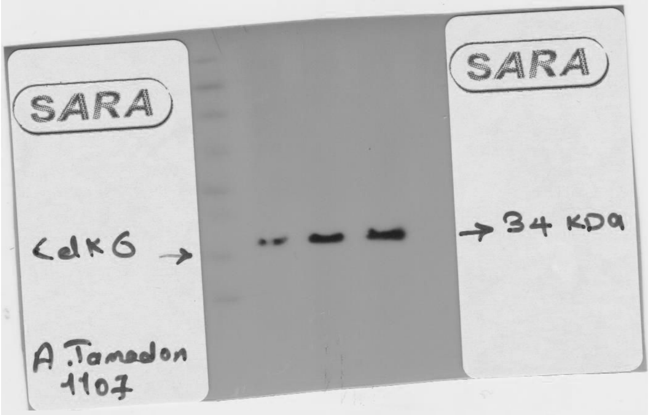
SARA
SARA
cdk6 →
→ 34 KDa
A. Tamadon
1107

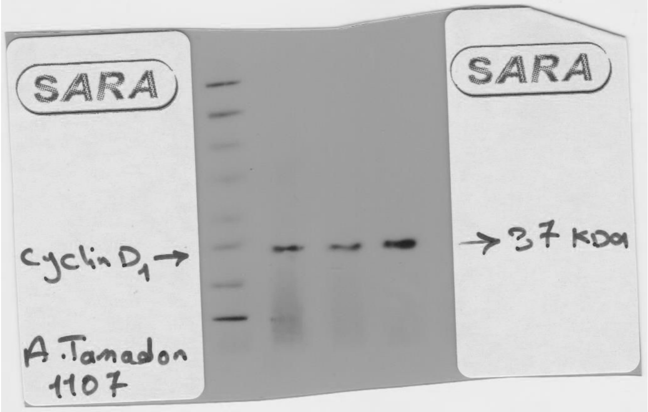
SARA
SARA
cyclin D1 →
→ 37 KDa
A. Tamadon
1107

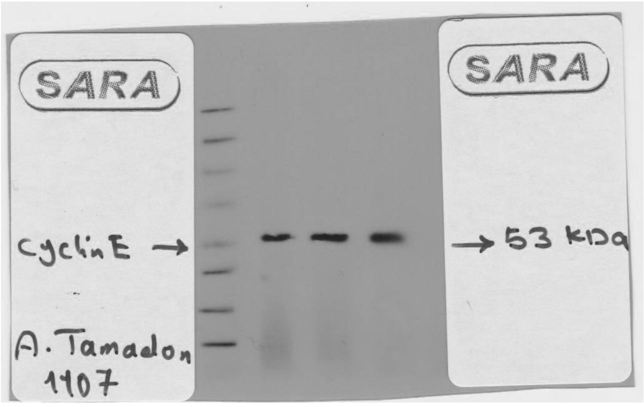
SARA
SARA
cyclin E →
→ 53 KDa
A. Tamadon
1107

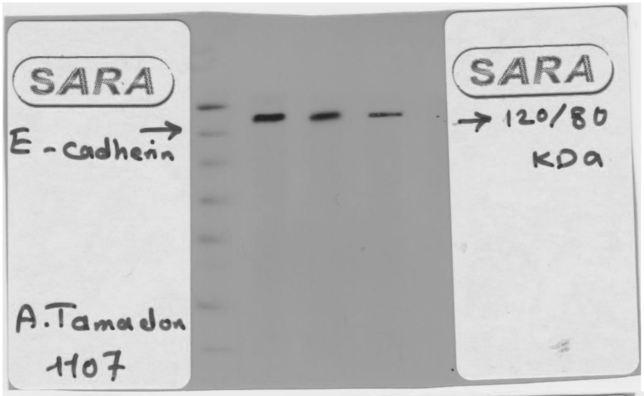
SARA
E-cadherin
A.Tamadon
1107
SARA
120/80
KDa

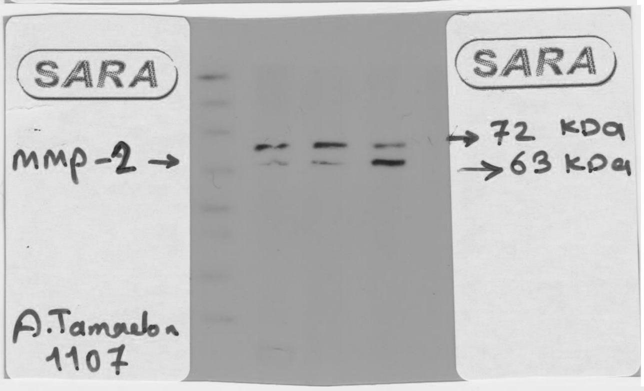
SARA
MMP-2
A.Tamadon
1107
SARA
72 KDa
63 KDa

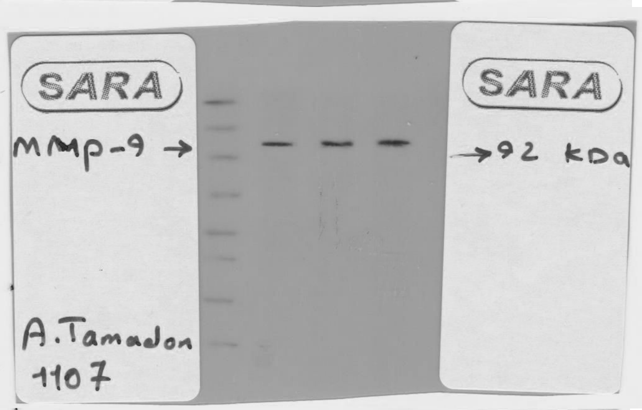
SARA
MMP-9
A.Tamadon
1107
SARA
92 KDa

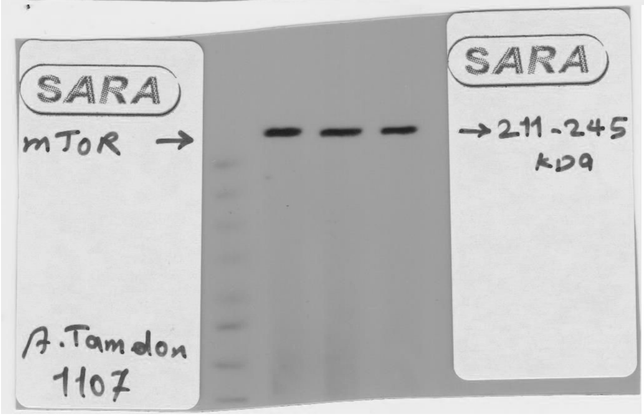
SARA
mTOR
A.Tamdon
1107
SARA
211-245
KDa

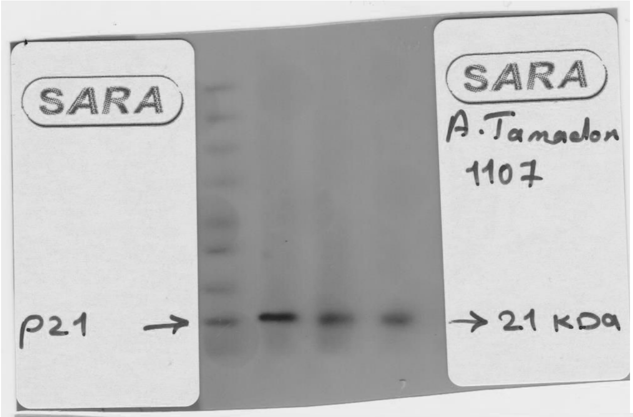
SARA
SARA
A. Tamadon
1107
p21 →
→ 21 KDa

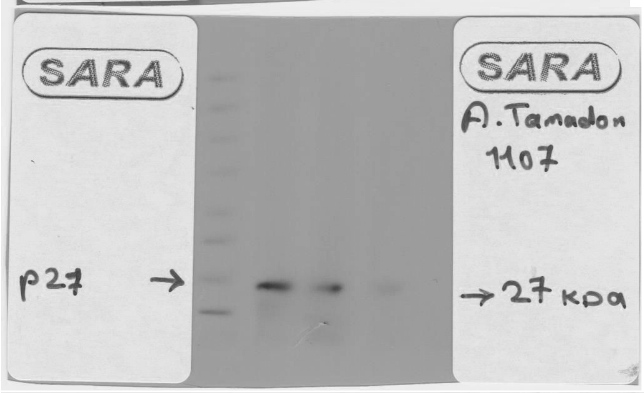
SARA
SARA
A. Tamadon
1107
p27 →
→ 27 KDa

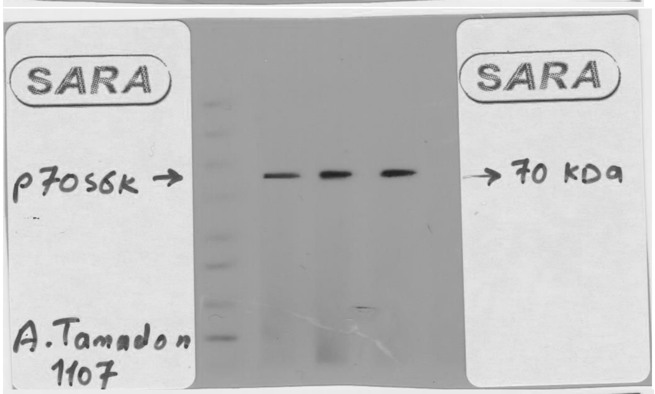
SARA
SARA
p70S6K →
→ 70 KDa
A. Tamadon
1107

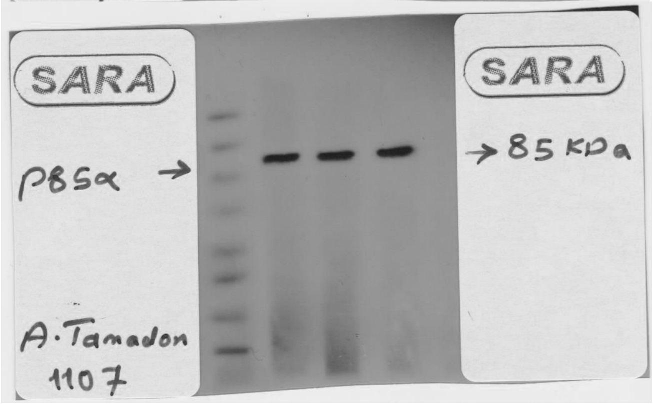
SARA
SARA
p85α →
→ 85 KDa
A. Tamadon
1107

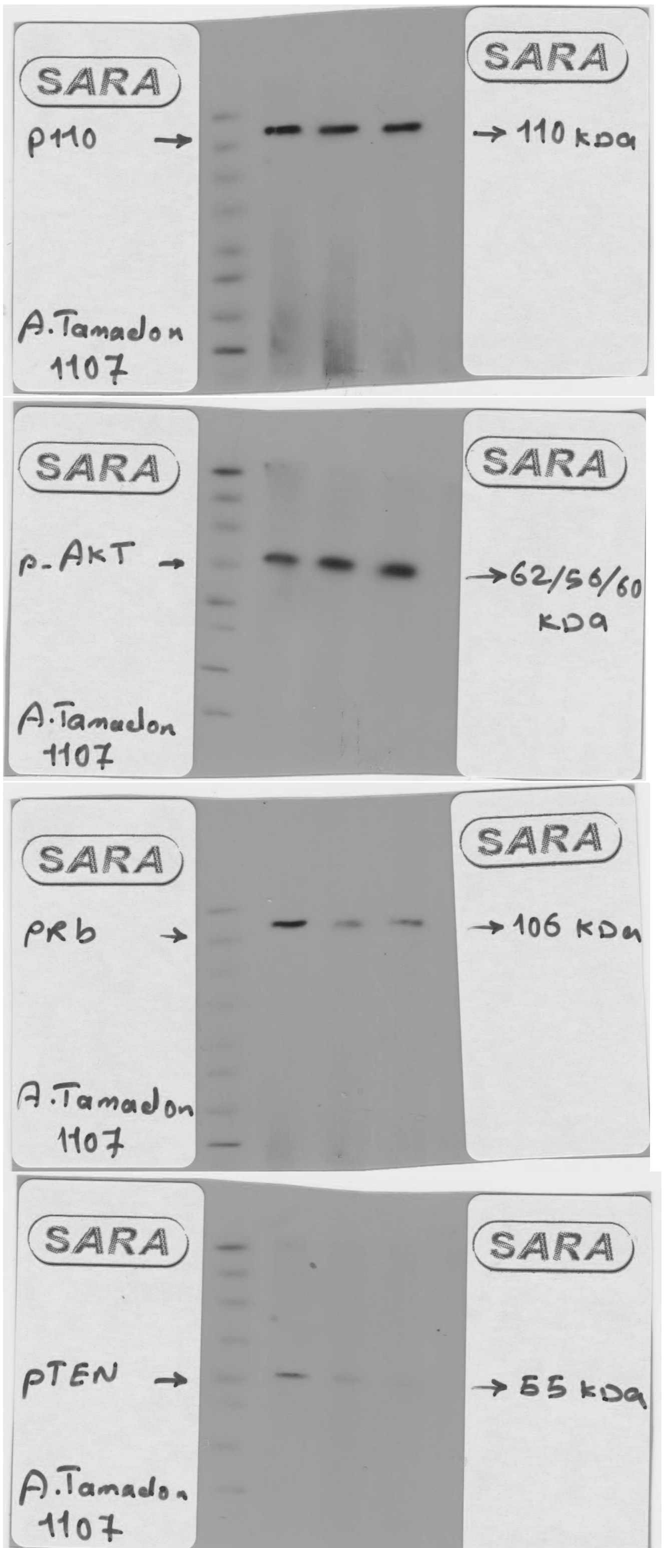

Figure S8. The original gels of western blot analysis
